# Delivery of a novel intervention to facilitate liberation from mechanical ventilation in paediatric intensive care: A process evaluation

**DOI:** 10.1371/journal.pone.0293063

**Published:** 2023-11-27

**Authors:** Joanne Jordan, Lyvonne Tume, Mike Clarke, Danny McAuley, Cliona McDowell, Lisa McIlmurray, Kevin Morris, Mark Peters, Timothy Walsh, Bronagh Blackwood

**Affiliations:** 1 School of Health, Wellbeing and Social Care, Open University, Milton Keynes, England; 2 Faculty of Heath, Social Care & Medicine, Edge Hill University, Ormskirk, England; 3 PICU, Alder Hey Children’s NHS Trust, Liverpool, England; 4 Centre for Public Health, School of Medicine, Dentistry and Biomedical Sciences, Queen’s University Belfast, Belfast, Northern Ireland; 5 Wellcome-Wolfson Institute for Experimental Medicine, Queen’s University Belfast, Belfast, Northern Ireland; 6 Northern Ireland Clinical Trials Unit, Royal Hospitals, Belfast, Northern Ireland; 7 Birmingham Women’s and Children’s NHS Foundation Trust, Birmingham, England; 8 Institute of Applied Health Research, University of Birmingham, Birmingham, England; 9 Great Ormond Street Institute of Child Health, NIHR Biomedical Research Centre, University College London, London, England; 10 Great Ormond Street Hospital, London, England; 11 Institute of Population Health Sciences, University of Edinburgh, Edinburgh, Scotland; Stellenbosch University Faculty of Medicine and Health Sciences, SOUTH AFRICA

## Abstract

**Background:**

Prolonged mechanical ventilation increases the risk of mortality and morbidity. Optimising sedation and early testing for possible liberation from invasive mechanical ventilation (IMV) has been shown to reduce time on the ventilator. Alongside a multicentre trial of sedation and ventilation weaning, we conducted a mixed method process evaluation to understand how the intervention content and delivery was linked to trial outcomes.

**Methods:**

10,495 children admitted to 18 paediatric intensive care units (ICUs) in the United Kingdom participated in a stepped-wedge, cluster randomised controlled trial, with 1955 clinical staff trained to deliver the intervention. The intervention comprised assessment and optimisation of sedation levels, and bedside screening of respiratory parameters to indicate readiness for a spontaneous breathing trial prior to liberation from ventilation. 193 clinical staff were interviewed towards the end of the trial. Interview data were thematically analysed, and quantitative adherence data were analysed using descriptive statistics.

**Results:**

The intervention led to a reduced duration of IMV (adjusted median difference– 7.1 hours, 95% CI -9.6 to -5.3, *p* = 0.01). Overall intervention adherence was 75% (range 59–85%). Ease and flexibility of the intervention promoted it use; designated responsibilities, explicit pathways of decision-making and a shared language for communication fostered proactivity and consistency towards extubation. Delivery of the intervention was hindered by established hospital and unit organisational and patient care routines, clinician preference and absence of clinical leadership.

**Conclusions:**

The SANDWICH trial showed a significant, although small, reduction in duration of IMV. Findings suggest that greater direction in decision-making pathways, robust embedment of new practice in unit routine, and capitalising on the skills of Advanced Nurse Practitioners and physiotherapists would have contributed to greater intervention effect.

**Trial registration:**

isrctn.org Identifier: ISRCTN16998143.

## Introduction

This paper reports the findings of a process evaluation conducted alongside a pragmatic clinical trial that evaluated a behaviour-change intervention designed to expedite liberation from mechanical ventilation for critically ill children in the intensive care unit (ICU). The intervention was complex including several interacting components [1]. All components had to be delivered daily by the multi-disciplinary clinical team and required clinicians to change from their usual practice and adopt the new intervention. The intervention was evaluated in 18 UK paediatric ICUs of various sizes, resources and organisational cultures. In such multicentre trials, an assessment of the primary outcome may provide an answer to the research question ‘does it work?’ (in this case, to reduce the duration of time on the ventilator), but does not consider ‘how’ this was achieved.

Process evaluations provide important insights into how complex interventions do or do not produce change. This is achieved by studying the processes of intervention implementation, receipt and delivery [1, 2] that involve collecting measures of intervention fidelity (delivered as planned), dose (proportion delivered) and reach (proportion of eligible patients receiving the intervention). Process evaluations also investigate contextual issues impacting delivery that include participant understanding and response, organisational norms and working, and available resources [3, 4]. By showing how an intervention works in everyday clinical settings, process findings can be used to guide adoption into real-world practice [5, 6]. Given the focus on practical potential, process evaluations are of particular value in pragmatic trials [7].

### The sedation and weaning in children (SANDWICH) trial

The **S**edation **and W**eaning **i**n **Ch**ildren (SANDWICH) trial was a stepped wedge, cluster randomised controlled trial that investigated if a ventilation liberation intervention reduced the duration of invasive mechanical ventilation (IMV) in children [8]. The intervention was evidence-based, drawing on two Cochrane systematic reviews [9, 10] and designed to capitalise on bedside nurses’ involvement to maximise clinical team engagement. A comprehensive education package was delivered to all multidisciplinary staff in all sites using a multi-faceted approach that included both on-line and face-to-face engagement [11]. The intervention permitted flexibility in delivery, while maintaining the core components. The comparator was usual care (Table 1).

**Table 1:** SANDWICH and usual care.

SANDWICH	Usual care
Multidisciplinary ward round, review of patient sedation assessment (COMFORT values) and ventilation criteria (daily screening), and daily targets fed back to the bedside nurse	Mainly medical disciplines. Sedation and ventilator weaning decisions not guided by COMFORT or daily ventilation assessments
Guidelines for nurse management of sedation optimisation involving 6-hourly COMFORT assessment and titration of sedatives to achieve the optimal target range	COMFORT assessment by nurses at variable time points No sedation optimisation guide
Twice daily screen of 5 ventilation parameters incorporating a higher respiratory support parameter to trigger earlier readiness to undertake a spontaneous breathing trial. A spontaneous breathing trial (SBT) on a low level of respiratory support to test extubation readiness.	Mainly small reductions in respiratory support over variable time periods to very low levels of respiratory support before testing readiness to extubate No or minimal nursing involvement

COMFORT is the name of the tool used to assess sedation and comfort of infants and children in paediatric intensive care. A full description of the intervention using the TIDieR checklist is available [12].

**Fig 1:**
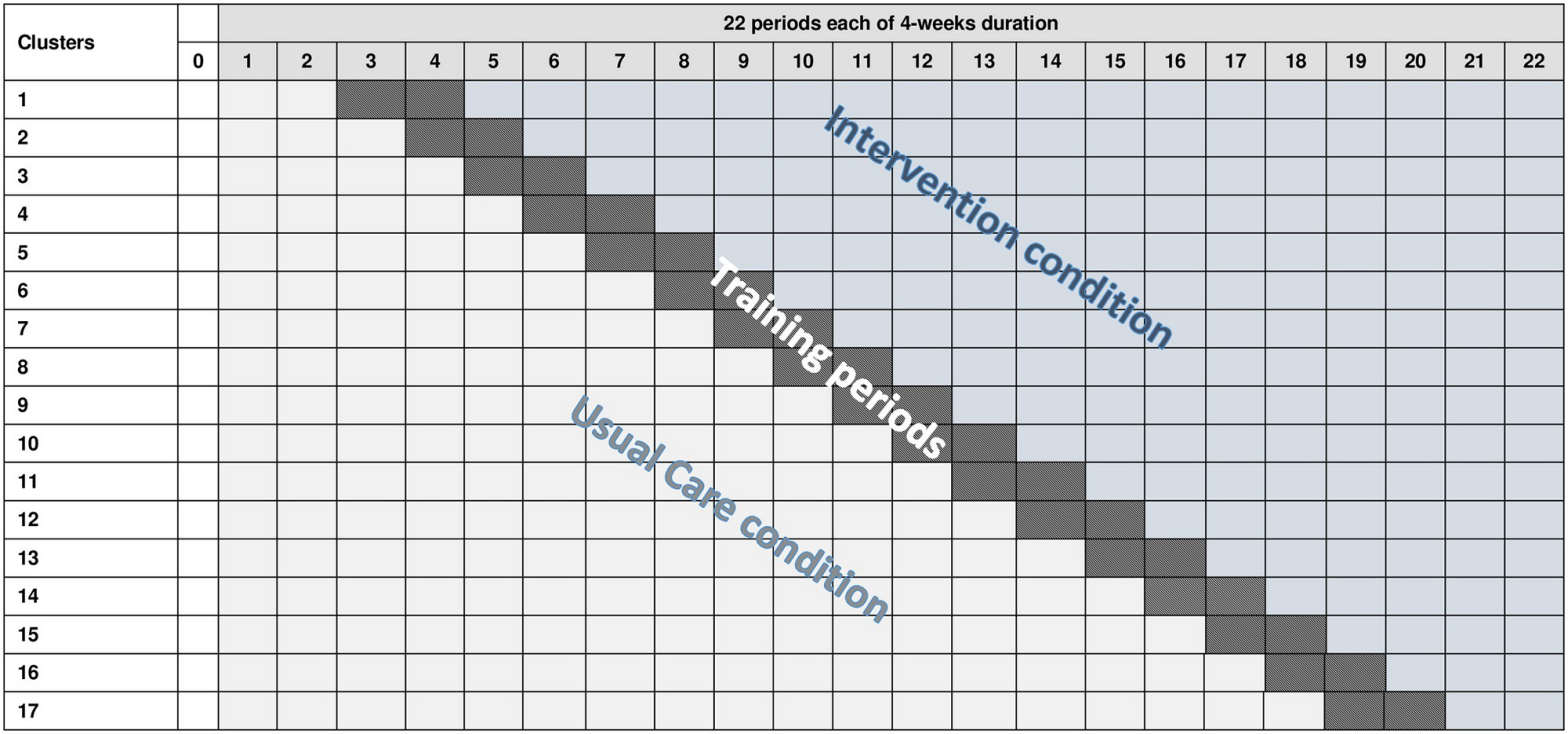
The SANDWICH trial schematic.

The stepped wedge trial had 22 time periods; each period duration was four weeks. All participating ICUs started in the usual care period, and one ICU was sequentially randomised every four weeks to enter an eight week training period, before crossing over to the intervention period for the remainder of the trial (Fig 1). The trial primary outcome was duration of time from intubation to first successful extubation.

The process evaluation was conducted to aid understanding of delivery of the intervention. The Medical Research Council (MRC) guidance on process evaluations [1] differentiates between *what* is delivered and *how* delivery is achieved. In this paper, we focus on the latter. In doing so, we address the following research question: ‘How did the processes involved in intervention delivery impact on the outcome of the trial?’

## Methods

### Aim and objectives

The aim of the study was to determine how the processes involved in intervention delivery affected the outcome of the trial. The objectives were to determine the fidelity, dose and reach of the intervention, and the factors and processes involved in delivery of the intervention.

### Design

**Fig 2:**
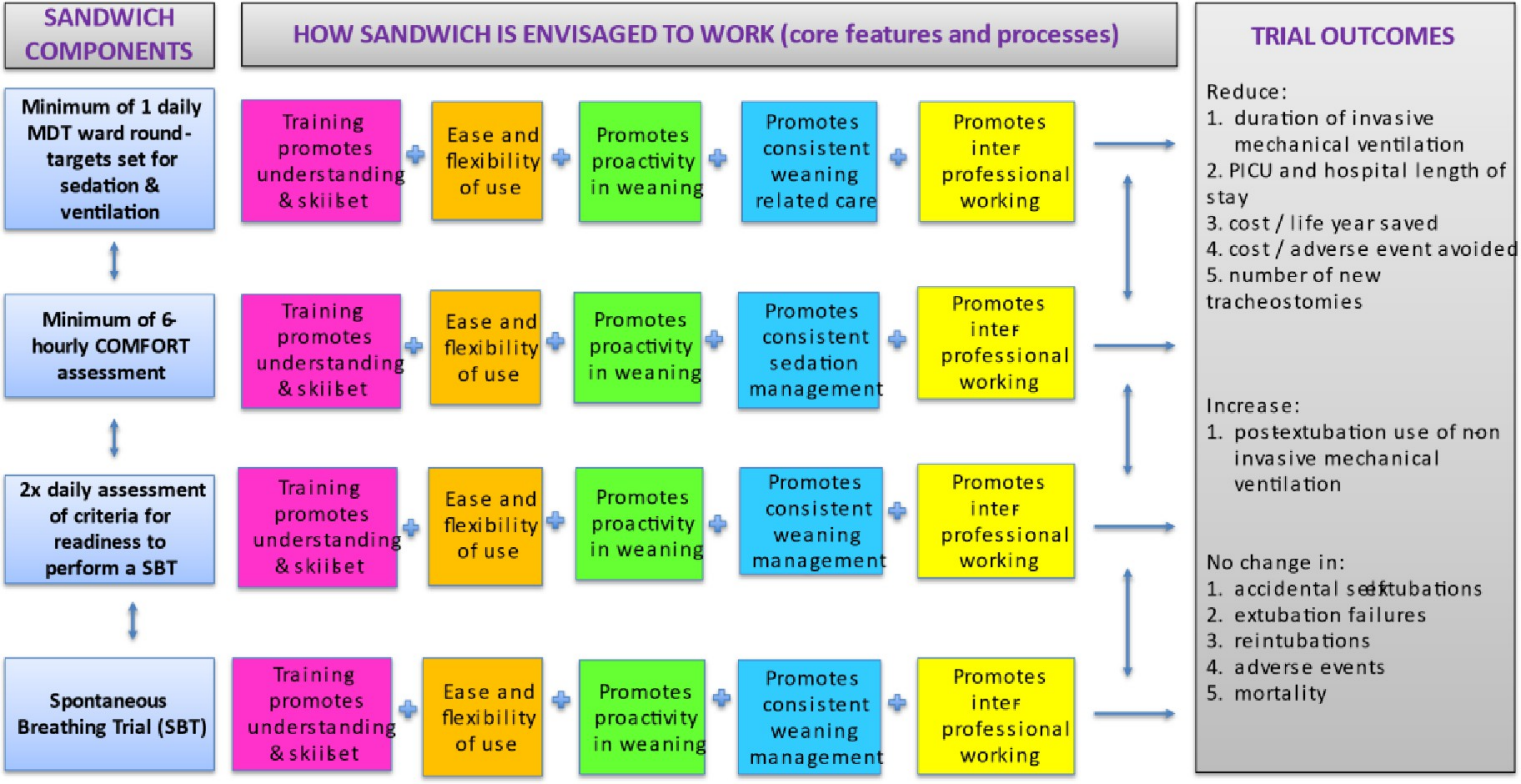
SANDWICH logic model. Adapted from Blackwood et al., 2022 [14]. COMFORT is the name of the tool used to assess sedation and comfort of infants and children in paediatric intensive care. Abbreviations: MDT, multidisciplinary team; PICU, paediatric intensive care unit; SBT, spontaneous breathing trial.

This was a process evaluation using mixed methods. We developed a logic model for the SANDWICH intervention, based on our earlier Cochrane review [13] and extended expert discussion [S1 Appendix]. The logic model specified the underpinning causal assumptions of the intervention, setting out its main components and the five processes by which they were envisaged to work together to achieve trial outcomes (Fig 2). The process evaluation investigated these processes to consider the extent to which they operated as intended and potential impact on study outcomes. The clinical trial and process evaluation study was approved by the National Research Ethics Committee East Midlands (reference 17/EM/0301) on 12 September 2017. We used the MRC guidance for process evaluations of complex interventions [1] and the Standards for Reporting Qualitative Research (SRQR) checklist [14] for reporting qualitative research [S2 Appendix].

### Data collection and analysis

#### Quantitative data collection

Data collected to determine the fidelity, dose and reach of the intervention included:

1. Daily adherence to conducting the intervention components (multidisciplinary ward round; COMFORT assessment; screening for readiness for a spontaneous breathing trial; undertaking an SBT when screening criteria were met)
2. Numbers of staff trained in the intervention
3. Numbers of children admitted requiring IMV and numbers screened for trial eligibility

Trained research nurses collected and recorded the daily adherence data onto case report forms (CRF). Staff training data were recorded on study training logs, and admission and recruitment data were collected from the study recruitment logs, and the paediatric intensive care national database.

#### Quantitative data analysis

Adherence was measured by the proportion of each intervention component performed and captured daily; the number of staff trained; and intervention reach (admissions screened divided by IMV admissions during the trial period). Due to sequential recruitment of clusters over time, we did not measure adherence over time, but provide the final mean adherence proportions for each ICU, ranked from highest to lowest. The trial’s primary outcome was the duration of IMV, measured from initiation of ventilation to the first successful extubation event. The estimate of the treatment effect was a time and cluster adjusted hazard ratio (HR) with 95% confidence intervals. The duration of IMV was analysed using a Cox proportional hazards model, with a frailty term for clustering by ICU. The time to event outcome was censored at the transition from usual care to the intervention training period, hospital discharge, 90-days, death, or if the child received a tracheostomy. We derived an absolute measure of effect by calculating the median of the model-based prediction of survival duration at all 22 periods in the stepped wedge trial and the difference between the intervention and usual care periods, and we summarised the extent of variability over the 22 periods using the interquartile range.

#### Qualitative data collection

A senior ethnographer (JJ), who was not involved in the clinical trial, visited participating sites to undertake face-to-face individual and focus group interviews. All clinical staff involved in delivering the intervention were eligible for interviews. Recruitment entailed an initial email from the researcher comprising an introduction, with attached Participant Information Sheet (PIS). The email was distributed to all eligible staff ahead of site visits. From amongst the staff working on the days of site visits, a range of clinicians and grades were purposively selected for interview. Prior to the interview, participants were given an opportunity to read the PIS again and ask questions. Once satisfied that informed consent had been completed, participants were asked to sign a Consent Form.

No prior relationship between JJ and participating staff existed, and all were made aware of her impartiality and need to gain candid insight into their experiences of trial delivery. Guided by a semi-structured interview schedule, discussions explored participant understanding and experience in relation to the five areas theorised as underpinning intervention effectiveness: training; flexibility; proactivity; consistent weaning and sedation care and management; and MDT working. The interview guide was developed by JJ and the Chief Investigator (BB). It was piloted with another author (LMcI), who is a paediatric intensive care nurse. Interviews were undertaken in a quiet location close to the paediatric ICU, lasting between 30–90 minutes. They were audio-recorded and fully transcribed, with anonymisation. Only JJ had access to information that could identify individual participants during and after data collection.

#### Qualitative data analysis

Data were analysed deductively and inductively. Deductively, the four components of the intervention and associated processes were used as a ‘point of entry’ into data. Inductively, Braun and Clarke’s thematic content analysis framework [15] was used to generate themes that crosscut the entirety of the qualitative dataset [S3 Appendix]. Individual and group interview data were combined [16, 17]. The same ethnographer led data analysis. To promote confirmability and trustworthiness, a 15% sample of data was sent to an independent qualitative researcher (CK) to check the analysis, identifying key differences and similarities in pursuit of an agreed final analysis [18]. Interview findings are presented according to the five proposed processes of how the intervention was envisaged to achieve the trial outcome outlined in the logic model.

## Findings

The patient recruitment period in the clinical trial was from February 2018 to October 2019 and last patient follow up was November 2019. The recruitment period for the process evaluation was from February 2018 to March 2020.

In total, 10,495 admissions to 18 paediatric ICUs were analysed. Findings for all ventilated children showed a significant reduction of 7.1 hours median difference (IQR -9.6 to -5.3, p = 0.01) between usual practice and the intervention in favour of the SANDWICH intervention. Further detailed results from the clinical trial are published elsewhere [11, 19].

### Fidelity, dose and reach of the SANDWICH intervention

Across the paediatric ICUs, the intervention reached a high proportion of patients (median 82%, IQR 77%, 89%). The median (IQR) of percentage adherence to the intervention components across the paediatric ICUs was highest for setting targets on the ward round (ventilation, 90% [QR 81, 96]; sedation, 85% [IQR 63, 89]) and COMFORT assessment (83% [IQR 82, 91]); moderate for daily screening (74% [IQR 66, 83]) and lowest for undertaking a SBT when criteria were met (40% [IQR 31, 51]). The mean of all components was 75% (range 59–85%). The proportion of intervention adherence within each ICU is presented in Table 2.

**Table 2:** The proportion (%) of intervention adherence in each paediatric intensive care unit.

Paediatric ICU ID	COMFORT assessed	Target set COMFORT	Target set ventilation	Readiness for SBT assessed	SBT initiated	Training target	Reach^1^	Average
16	93.5	98.3	99.1	81.5	54.8	82	88.6	85.4
17	88.0	92.0	96.0	70.8	60.0	100	82.3	84.2
18	90.9	93.5	95.9	84.2	53.0	85	74.5	82.4
15	96.9	96.5	98.4	63.3	51.9	76	76.7	80.0
02	84.1	89.1	91.6	66.0	48.4	80	92.5	78.8
01	82.0	87.4	87.3	89.9	21.6	90	88.3	78.1
13	81.6	89.3	91.8	74.5	38.5	84	86.8	78.1
08/09^2^	84.7	81.5	89.95	62.6	39.2	94	86.6	76.95
06	69.8	65.2	95.0	81.0	50.6	90	82.3	76.3
05	90.7	87.1	81.4	54.0	32.0	81	100.0	75.2
12	83.1	82.4	82.6	72.5	33.1	86	78.0	74.0
04	79.3	62.5	88.0	61.3	45.4	91	89.5	73.9
11	75.8	77.5	73.6	86.8	29.5	83	71.9	71.2
07	77.5	63.2	96.3	76.1	19.8	80	75.3	69.7
10	94.0	34.8	34.8	92.5	44.7	78	72.6	64.5
03	81.8	53.7	56.8	83.4	15.0	59	85.3	62.1
14	83.2	39.1	28.1	66.8	30.8	86	80.1	59.2

^1^Reach was defined as the extent to which a target population came into contact with the intervention, measured by the proportion of patients screened (recruits and exclusions) divided by IMV admission patients.

^2^Two paediatric ICUs in the same hospital were randomised together and analysed as one site.

### Interview participants

In total, 1,955 staff from 18 paediatric ICUs were trained in the intervention and 193 of these staff participated in end of trial interviews. They included 112 nurses (bedside, senior clinical management, and advanced nurse practitioners); 42 medical staff (medical trainees and consultant intensivists); 14 allied health professionals (physiotherapists, pharmacists and nursing assistants); and 25 research team members.

### Factors and processes involved in delivery of the intervention

Supporting quotes for the following analysis are presented in S4 Appendix.

#### Training promotes understanding and skill set

Overall, the training was considered integral to staff understanding of the purpose and content of the intervention and the effective discharge of their respective roles and responsibilities. Training was regularly discussed as time-consuming, particularly the online component. Face-to-face training was considered more enjoyable and effective due to its practical nature and focus, including as participants could ask questions and resolve emerging issues. In contrast, the online component could be described as lacking an immediate “real-world” relevance.

### Ease and flexibility of use

#### Intervention acceptability

The intervention was consistently validated as technically straightforward to use and accommodate within routines of care. Although bedside nurses were aware of increased documentation, overall, they endorsed the ease and flexibility with which COMFORT scoring and SBT readiness screening could be undertaken. That the intervention explicitly accommodated independent clinical decision-making was considered particularly important for buy-in from unit consultants, who expressed no sense of being constrained in their decision-making regarding either the conduct of SBTs or patient extubation. The same independence was highlighted by nursing staff, primarily in relation to the management of patient sedation.

#### Stasis in the absence of explicit direction

Although the intervention’s strategic flexibility was widely endorsed, it was also discussed as inadvertently working against optimal intervention delivery. Three main issues were identified. First, the flexibility inherent in the COMFORT assessment, which was categorised into a range of scores indicating sedation ‘status’ for weaning. The categorisation could be problematic due to an overlap in ranges (10–12 consider weaning sedation; 12–17 comfortable and can be safely extubated). A child who scored in the range 12, or just above, could fail a SBT screen and remain longer on the ventilator. Second, the option of running a SBT for up to two hours. This period of time could allow attention to move away from a patient’s weaning progress as other priorities of patient care took precedence. It could also diminish the perceived importance of the need to extubate, as staff were aware a patient could remain on SBT settings for two hours. On the occasions when a patient remained on a SBT for longer than two hours, the end goal of extubation could be undermined in that the patient could tire and need to be returned to higher levels of support. Third, the intervention did not stipulate that extubation should follow within a set time after a successful SBT, in recognition that this might not be practically possible. However, the lack of such a requirement was linked to an indefinite postponement of extubation in some cases as other unit pressures took priority and/or relevant staff were unavailable.

#### Promotes proactivity in weaning

*Effectiveness of ward round planning*. The morning ward round was the primary forum of decision-making regarding daily plans for individual patient care in all units. Building on this opportunity, the intervention included explicit guidance on the use of ward rounds to set targets for patient sedation and weaning. Where discussion occurred, participants gained clarity concerning plans. Conversely, deficits in ward round discussion, and thus planning, were associated with uncertainty and potential delay in patient weaning progress. Overall, accounts suggested inconsistent adherence both within and between units. Three main factors were implicated. First, that those leading the ward round ensured relevant discussion took place. Second, discussion was more likely to occur if a patient was considered to be approaching readiness for extubation. Third, weaning plans could be de-prioritised if other information took precedence within a tight time frame for discussion.

*Development of an underpinning momentum towards extubation*. To varying degrees, participants in all units considered adoption of the intervention to have encouraged an overall momentum towards patient extubation. Several inter-related processes were identified. First, staff were prompted to consider weaning and keep opportunities for progress at the forefront of thinking. Second, staff were enabled to advance weaning according to their respective roles and responsibilities without explicit direction. Third, inconsistency was reduced as staff who might otherwise adopt a more extended approach to weaning were discouraged from doing so. Finally, anticipatory patient preparation was encouraged in respect of what might ‘come next,’ e.g. stopping patient feeds ahead of a SBT and potential extubation.

#### Promotes consistent weaning related care

*Enhanced prioritisation and conduct of patient sedation*. Across all professions, participants upheld the positive impact of COMFORT assessment in promoting appropriate and consistent sedation management. Consistency was linked to the shared multidisciplinary language provided by the intervention. Resulting improvements included increased attention to other factors impacting on patient comfort (e.g. pain, and improved drug regimes), which were associated with enhancing extubation readiness.

*Impulse to adhere to preferred sedation practice*. Inconsistencies in the performance of COMFORT assessment and attendant sedation management were associated with two issues. First, the prioritisation of other care, particularly in the context of heavy workloads and unwell patients. Second, a preference amongst bedside nurses, particularly the inexperienced, to maintain patient safety by keeping children ‘well’ sedated. The intervention itself could be implicated in over-sedation in that it could be followed ‘technically’, but still allow for a child to remain too well sedated.

*Expediting patient extubation*. Participants confirmed that units incorporated SBTs with daily readiness screens into their practice, indicating that both were valuable in advancing extubation. Units created times for both activities to fit with established routines. SBT readiness screens scheduled for the early morning, typically towards the end of the night shift, were described as most consistently undertaken for two reasons: night nurses had the requisite time available, and it ensured the result was available for the morning ward round discussion. Compared to SBTs scheduled for later in the day, participants confirmed that those scheduled for the morning were also more consistently performed and led to patient extubation due to better staffing resources and established routines of patient care.

Both medical and nursing staff discussed the valuable learning gained concerning the validity of the SBT readiness screen/SBT for advancing patient weaning. Overall, this involved acceptance of the value of moving away from a gradual “step-down” approach to one of weaning patients more dynamically. Thus, one of the most frequently articulated aspects of learning concerned the potential for weaning to be safely expedited by taking patients from a relatively high pressure support to a lower pressure support during the SBT.

*Impulse to adhere to preferred ventilator weaning practice*. Participants highlighted the persistence of established practice, resulting in inconsistent intervention adherence and delayed weaning progression. Various manifestations were described. Doctors could insist on personally assessing all patients before moving to the ‘next step’ of the intervention. They could also continue to wean patients onto progressively lower ventilator settings before extubation, rather than initiate a SBT following a successful screen. In some cases, SBTs were performed only when low ventilator settings had been reached. In such cases, rather than being used to potentially expedite extubation, the SBT was being used as a final check. Following a successful SBT, but where extubation was contraindicated, on occasions, patients were returned to full ventilator support due to consultant aversion to leaving patients on low pressure support for any length of time. Finally, inexperienced medical and nursing staff were sometimes described as prematurely ending a SBT based on initial changes in a patient’s breathing rate, which may have settled as patients became accustomed to the new mode/level of support. Where non-adherence to the protocol occurred, doctors were required to provide a rationale for their decision-making. Bedside nurses, who were responsible for recording the reasons, could express frustration that none was given or that adequate detail was missing.

*Variability of consistency relating to unit routines*. Participants highlighted opportunities and challenges to consistent intervention delivery afforded by the daily routines of unit activity. One challenge concerned the morning ‘ward round’. In many units, off-unit rounds took place involving the medical team and senior nurse-in-charge, with the latter communicating plans ‘back’ to bedside staff. This practice was considered to negatively affect full multidisciplinary weaning decision-making. Potentially, relevant patient information might not be communicated, and decisions could be made without input from bedside nurses. Senior nurses highlighted frustration at being unable to achieve proper trial-related discussions during off-unit handovers, meaning they had to make plans based on non-specific discussions.

Many accounts suggested a diminished impetus to undertake SBT screening and SBTs in late afternoon or evening because unit routine practice meant patients were less likely to be extubated. Even when units operated with a formal ‘24/7’ extubation policy, participants acknowledged that policy implementation was selective. For example, if patients passed a SBT later in the day, ventilator pressures were reduced, but patients often remained intubated until a decision the next morning. Thus, instead of expediting ventilator liberation, the function of later screening and SBTs was seen as providing insight into patient progress.

Patient weaning or extubation could be subordinated to other core unit activity, resulting in patients remaining on ventilation for several hours. Examples included patient investigations or clinical procedures, off-unit investigations, and staff breaks. In some units, participants described a routine of stopping feeds in the early morning (typically 4 hours) in anticipation of a successful SBT as contributing to timely extubation. Conversely, in units where this routine was absent, extubation could not immediately follow a successful SBT because the feed-stopping period had not commenced.

Intervention adherence was most strenuously questioned when it was understood to be largely redundant in terms of patient care. For example, participants talked about non-performance of SBT and screening when staff were aware that patients were clinically inappropriate for extubation. Similarly, ward round discussions of ventilator and sedation targets more likely occurred when a patient was approaching readiness for extubation. An awareness of the adverse impact of such behaviour on trial delivery was often accompanied by avowals that selective performance of intervention-related tasks was rational, especially in the context of an already heavy workload. The tailoring of trial delivery to maximise relevance, as well as the possibilities for timely extubation, was commonly described.

*Availability of appropriately skilled staff*. The impact of the availability of medical consultants on weaning progress was repeatedly discussed by participants. Across all units, when the consultant was absent, responsibility for patient care fell to less experienced medical colleagues. Participants highlighted delays in weaning progression during such times, based on a combination of personal lack of confidence in their knowledge and skills, or fundamental disinterest amongst these staff; lack of confidence in their skill set on the part of senior colleagues; and the 3–6 monthly rotation of this staff group militating against relevant training in trial initiatives. A frequently described example of delayed weaning progress concerned their refusal to undertake a SBT until consultants were available.

Some units employed Advanced Nurse Practitioners (ANPs) with specialist training in patient weaning and extubation. At times, participants described the ANPs as central to the ability of the unit to progress weaning/extubation in the absence of consultant cover. In units where ANPs did not use these skills, a missed opportunity for trial delivery was highlighted. In some units, more experienced bedside nurses who had completed an advanced training course were authorised to perform SBTs, thereby increasing the availability of staff able to progress weaning. Differential physiotherapy involvement in weaning meant that only a few units authorised physiotherapists to extubate. In these units, physiotherapist participants highlighted the advantages of their involvement in terms of availability of staff to execute SBTs and/or extubate in the absence of consultant cover.

In some units, staff recruitment issues impacted trial delivery. A lack of a full complement of consultants, and/or frequent changes to the medical team, were discussed as increasing consultant workload. This engendered a concentration on priorities of patient care to the detriment of trial related activity. Additionally, locum consultants employed to make up staff shortfalls were inevitably less versed in trial activity and likely to bring greater diversity in terms of individual practice. Problems with nursing recruitment and retention were also frequently identified. Many units were dependent on junior nurses or bank and/or agency nurses. Lack of confidence and/or a preoccupation with basic patient care meant these nurses were likely to be less engaged in SANDWICH.

#### Promotes inter-professional working

Across all disciplines of participants, there was widespread agreement that the trial improved inter-professional working by: broadening the range of staff involved in sedation and weaning management; outlining key roles to these different staff; and providing a shared language for communication. Of particular importance was the enhanced role played by bedside nurses. Throughout, the crucial factor identified was the assignment of specific roles and the shared decision-making through which these roles could be discharged. Both COMFORT scoring and SBT screening criteria were consistently discussed as having promoted bedside nurse autonomous practice, as well as their engagement in meaningful discussion about this practice with colleagues. In most units, the SBT screen gave bedside nurses a designated role in ventilator weaning for the first time. Bedside nurses were aware of this greater involvement, as they talked about more senior colleagues actively seeking out their recommendations for patient care. Throughout, there was a clear sense that the intervention had enhanced colleagues’ confidence in these recommendations because they were premised on a shared evidence base.

A similar, but more understated, enhanced confidence amongst senior nursing staff was apparent. They considered themselves the “drivers”, but not technical implementers, of weaning and saw their assigned SANDWICH role (to commence a patient on a SBT) to have the potential to become embedded as part of standard unit practice. Such a role was seen as representing an important advancement of their professional authority. Amongst physiotherapists, the trial was seen as having facilitated greater involvement for their own team, as they learnt more about appropriate sedation, and contributed to the formulation of patient weaning plans. There was universal agreement amongst medical participants that the trial had enhanced nursing confidence and autonomy of practice, which was viewed as a positive development. At times, their reflections were couched in a strategic context of their vision for unit inter-professional working. At other times, accounts focused on an awareness that this working had improved precisely because they were required to reflect on, and account for, their clinical decision-making.

Although the positive impact of the SANDWICH trial on inter-professional working was widely endorsed, several important caveats were articulated. First, the full realisation of nurse autonomy ultimately depended on its validation by senior, especially medical, colleagues. This could involve consultant failure to communicate with bedside nurses and/or acceptance or rejection of information and/or advice given by them. In this context, consultant participants described differential confidence in nursing staff based on their perceived knowledge and expertise. At times, senior nurses were described as retaining control of patient care, thereby limiting the potential for junior colleagues to fully discharge their designated roles. Second, although experienced nurses could be self-assured and comfortable in their interactions with senior nursing and medical colleagues, this did not hold true of the profession more generally. Several bedside nurses commented on their own reticence. Failure on the part of nurses to question medical colleagues was seen as an impediment to consistency in intervention delivery in that it could result in the perpetuation of deviation from the protocol. A case in point concerns the recording of reasons for decisions taken regarding failure to perform a SBT when indicated. Although it was a requirement that doctors explicitly account for such non-performance, with bedside nurses being responsible for recording the reasons given, in practice this did not always happen. Although frustrated, nurses frequently commented that they did not feel able to press doctors to provide a rationale. More broadly, a culture of bedside nurses not being involved in any aspect of ventilator weaning could mean they were limited, even excluded, by others, and limit or exclude themselves, from discharging their trial roles.

*Final authority rests with consultant*. As foreshadowed in the preceding section, across all units, participant accounts underscored the final authority of consultants regarding patient care, and consequent vulnerability of the intervention to their own willingness to adhere to the protocol, and to support the same adherence by other staff. In some units, the medical team was described as greatly facilitating unit wide adherence by demonstrating manifest interest and support. In other units, the opposite was identified, with doctors being described as disinterested, even obstructive. In both contexts, as members of staff in positions of final authority, the approach of consultants was understood to have played an important role in either promoting or impeding the consistency with which a unit adhered to the protocol and delivered the intervention as intended.

## Discussion

### Summary of findings

The trial met its primary outcome, achieving a significant (if small) reduction in time to successful ventilator liberation. Although the intervention reached a high proportion of patients, delivery did not always encompass all components that were deemed necessary in progression towards extubation. This may explain the lack of effect between adherence rates and the primary outcome. The setting of sedation and ventilator support targets, as well as COMFORT assessment, achieved high adherence, and were important foundational work for intervention effectiveness. However, sedation targets could be inadequately explicit and/or focused, so that assessment did not always result in optimal patient sedation. Furthermore, setting sedation and ventilator targets did not guarantee that these targets were acted on. The same was apparent in respect of daily SBT screening. Although this component achieved relatively high adherence and successfully moved patients along the weaning pathway, some drop-off was clear and, again, the passing of a SBT screen did not always ensure the subsequent performance of an SBT. Completion of SBTs achieved the lowest adherence; non-performance represented a detrimental ‘stop’ in patients’ pathway through the intervention and, by extension, progress towards extubation. Nevertheless, despite variable component adherence rates, the intervention was delivered as a coherent bundle.

The process evaluation enabled a nuanced understanding of multiple, overlapping processes, operating at the individual, unit and wider institutional level, involved in the delivery of the intervention. Importantly, the evidence provides insight into these processes in respect of each of the intervention components. It also highlights the countervailing nature of their collective working to moderate intervention delivery as theorised in our logic model.

### Implications of the evidence for trial theory

An extensive body of research demonstrates the benefits of staff training in promoting effective delivery of novel ICU interventions [13, 20–24]. Relevant knowledge and skills, familiarity with the intervention, and ‘buy-in’ to its aims and purpose can all be enabled. The SANDWICH experience upholds the value of training in these respects. It also provides additional insight concerning the most appropriate form and means of delivery. Given the tendency for the online component to be experienced as ‘distant’ from everyday practice, training that promotes felt immediacy is suggested, including opportunities to address issues arising. Relevant efforts could include, for example, the incorporation of training within unit working routines, including those at the patient bedside [25].

Our theory prioritised intervention ease and flexibility of use, an approach consistent with the ICU literature showing the benefits of ready integration within unit routines [13, 22, 26, 27]. Following Hawe and colleagues [28, 29] Gessell et al. [30] distinguish between the ‘form’ and ‘function’ of an intervention. While the form may be adapted to the local context, the integrity of the function must be maintained. In the case of SANDWICH, although the strategic flexibility of the intervention facilitated its accommodation into unit routines of care, on occasions it may also have worked to delay progress towards extubation. This is particularly the case regarding the absence of time-bound instructions concerning the execution of SBTs, and follow-up action where relevant. Providing a more structured approach in terms of the pace at which SBTs should be completed, as well as how quickly successful SBTs should be followed by extubation, may have helped in this regard.

Development of the theory underpinning our intervention was based on an ICU specific evidence base [13], meaning that it was tailored to processes already shown as relevant to that environment. Tailoring of interventions to specific settings and target groups has been shown to be effective [31], although the latter Cochrane review showed the effect is variable and tends to be small to moderate. In this context, the SANDWICH trial suggests the need for a careful balance between tailoring and the imperative for change. In some respects, it was precisely because of the intervention’s flexibility that departure from anticipated delivery occurred.

The evidence largely supports our theoretical assumptions concerning how the intervention would work to encourage proactivity in weaning. Even where intervention deviations occurred, still the units were operating with a bundle of components, meaning that, even incrementally, an overall imperative towards weaning was sustained. Our evidence aligns with other work on the effectiveness of multifaceted ICU interventions [32–34]. That deficits in ward round planning were more likely in respect of patients approaching readiness for extubation, and that it could be subordinated to other patient care requirements, suggests a de-prioritisation of weaning within a hierarchy of patient care. An important countervailing force was the intervention’s creation of an underlying positive momentum, whereby the issue of weaning became more embedded in staff thinking, so that they were more likely to attend to relevant issues even if this did not happen precisely according to intervention guidance.

As anticipated, the intervention worked to promote inter-disciplinary collaboration. Although the challenges inherent in inter-professional working in ICU have been documented [35, 36], our evidence shows that collaboration was not only possible, but also contributed significantly to intervention delivery. Bedside nurses were key to patient weaning progress, not only in terms of sedation management and performance of SBT screens, but also in liaising with senior colleagues at crucial junctures of a patient’s pathway through the intervention. Here, the provision of a shared multidisciplinary language was pivotal; staff could talk to one another and make decisions in ways that made sense collectively. The potential for inter-professional collaboration in improving patient weaning aligns with the benefits for patient care identified by other evidence from intensive care [37–39]. The process evaluation demonstrated bedside nurses’ willingness and technical competence to assume an enhanced role in patient ventilator weaning. This enhanced role was central to a team-based approach that reduced duration of IMV.

In terms of consistency of weaning related care (both sedation and weaning management), the evidence concerning our theoretical assumptions is more ambivalent. The protocol did work to improve consistency of care. Two examples are bedside nurses’ use of lighter patient sedation, and doctors’ uptake of a more dynamic approach to lowering ventilator settings. However, the impetus to adhere to established practice also meant that bedside nurses continued to over-sedate, and doctors continued to wean ‘down’ to lower support. The challenges inherent in changing established practice within ICUs are well documented [22, 26, 40–45]. A narrower set of COMFORT scores may have encouraged more optimal sedation by nurses. The protocol accommodated the complexity of clinical decision-making; it is the case that consultant adherence increased over time as its effectiveness was demonstrated. A particularly intractable issue impacting consistency of care was availability of medical staff to undertake relevant tasks. In line with evidence of the benefits to be accrued from greater involvement of other suitably trained clinical staff in intensive care [46–48], our evidence upholds the value of ANPs and physiotherapists in mitigating the absence of medical cover.

### Study strengths and limitations

We followed recommended guidance and tailored the process evaluation to the trial, the intervention and the outcomes studied [49]. A major strength of the study was that we collected qualitative and quantitative data, which enabled the triangulation of findings [4]. Analysis was therefore enhanced by being able to consider the evidence collectively, exploring relationships between the different sets of data. Further, qualitative data were collected from all units, encompassing the views and experiences of all professional groupings involved in weaning. We used a convenience sample of interview participants, drawn from staff available on the days of site data collection. Staff availability on the day meant that we were unable to ensure inclusion of all relevant staff. In addition, staff were selected by the unit research teams, meaning that selection bias may be present, and we cannot comment on staff who declined participation or their reasons for so doing. Despite these limitations, there was strong consistency in the views and experiences across all units, boosting our confidence in the validity of the findings.

The MRC Guidance on the conduct of process evaluations recommends the use of theory as a means of testing and refinement [1]. We developed an evidence-based theoretical framework specific to our intervention. Using the classification set out by McIntyre et al. (2020) [50] we therefore developed a *program* theory; such theories are considered useful in terms of being testable but lack generalisability. Combining the process evaluation and trial adherence data allowed us to critically interrogate our theory in terms of establishing the fit between what we hypothesised would be the processes through which the intervention achieved its effect, and the actual processes involved. Although we found considerable fit, we also uncovered countervailing processes that mitigated intended delivery. Using this evidence, we have endeavoured to offer suggestions as to how relevant issues might be addressed, providing transferable insight into how intervention delivery might be optimised in similar contexts and settings.

### Implications for future research

Future research should examine sustainability of the intervention in participating sites. This may shed light on the active components that were deemed by clinicians to be the most effective.

## Conclusion

The SANDWICH trial showed a significant, albeit small, reduction in duration of IMV for children. The process evaluation showed that the intervention’s ease of use and flexibility facilitated its adoption. Provision of a common language, designated bedside nursing involvement, and clear pathways for clinical decision-making collectively worked to expedite extubation. To further enhance this momentum, findings suggest the benefits of more explicit direction in decision-making pathways, robustly embedding new practice as part of a collective endeavour within unit routines, and capitalising on the skills of ANPs and physiotherapists to include a greater role in patient extubation.

## Supporting information

S1 Appendix SANDWICH implementation. (DOCX)

S2 Appendix Standards for Reporting Qualitative Research (SRQR) checklist. (DOCX)

S3 Appendix Process of theme development (following Braun & Clarke, 2006). (DOCX)

S4 Appendix Supporting quotes. (DOCX)

S1 Checklist *PLOS ONE* clinical studies checklist. (DOCX)

## References

[1] Moore GF, Audrey S, Barker M, Bond L, Bonell C, Hardeman W, et al. Process evaluation of complex interventions: Medical Research Council guidance. BMJ. 2015;350:h1258. doi: 10.1136/bmj.h1258 25791983 PMC4366184

[2] Lockwood I, Walker RM, Latimer S, Chaboyer W, Cooke M, Gillespie BM. Process evaluations undertaken alongside randomised controlled trials in the hospital setting: A scoping review. Contemp Clin Trials Commun. 2022;26:100894. doi: 10.1016/j.conctc.2022.100894 36684693 PMC9846456

[3] Moore G, Audrey S, Barker M, Bond L, Bonell C, Cooper C, et al. Process evaluation in complex public health intervention studies: the need for guidance. J Epidemiol Community Health. 2014;68(2):101–2. doi: 10.1136/jech-2013-202869 24022816 PMC3892708

[4] Oakley A, Strange V, Bonell C, Allen E, Stephenson J, Team RS. Process evaluation in randomised controlled trials of complex interventions. BMJ. 2006;332(7538):413–6. doi: 10.1136/bmj.332.7538.413 16484270 PMC1370978

[5] Ford I, Norrie J. Pragmatic Trials. N Engl J Med. 2016;375(5):454–63. doi: 10.1056/NEJMra1510059 27518663

[6] Patsopoulos NA. A pragmatic view on pragmatic trials. Dialogues Clin Neurosci. 2011;13(2):217–24. doi: 10.31887/DCNS.2011.13.2/npatsopoulos 21842619 PMC3181997

[7] French C, Pinnock H, Forbes G, Skene I, Taylor SJC. Process evaluation within pragmatic randomised controlled trials: what is it, why is it done, and can we find it?-a systematic review. Trials. 2020;21(1):916. doi: 10.1186/s13063-020-04762-9 33168067 PMC7650157

[8] Blackwood B, Agus A, Boyle R, et al; Paediatric Intensive Care Society Study Group (PICS-SG). Sedation and Weaning in Children (SANDWICH): protocol for a cluster randomised stepped wedge trial. BMJ Open. 2019;9(11):e031630. doi: 10.1136/bmjopen-2019-031630 31712342 PMC6858098

[9] Blackwood B, Murray M, Chisakuta A, Cardwell CR, O’Halloran P. Protocolized versus non-protocolized weaning for reducing the duration of invasive mechanical ventilation in critically ill paediatric patients. Cochrane Database Syst Rev. 2013;2013(7):CD009082. doi: 10.1002/14651858.CD009082.pub2 23900725 PMC6517159

[10] Blackwood B, Burns KE, Cardwell CR, O’Halloran P. Protocolized versus non-protocolized weaning for reducing the duration of mechanical ventilation in critically ill adult patients. Cochrane Database Syst Rev. 2014;2014(11):CD006904. doi: 10.1002/14651858.CD006904.pub3 25375085 PMC6517015

[11] Blackwood B, Tume LN, Morris KP, Clarke M, McDowell C, Hemming K, et al. Effect of a Sedation and Ventilator Liberation Protocol vs Usual Care on Duration of Invasive Mechanical Ventilation in Pediatric Intensive Care Units: A Randomized Clinical Trial. JAMA. 2021;326(5):401–10. doi: 10.1001/jama.2021.10296 34342620 PMC8335576

[12] Tume LN, Blackwood B, McAuley DF, Morris K, Peters MJ, Jordan J, et al. Using the TIDieR checklist to describe the intervention of the Sedation and Weaning in Children (SANDWICH) trial. Nurs Crit Care. 2022. doi: 10.1111/nicc.12810 35733409

[13] Jordan J, Rose L, Dainty KN, Noyes J, Blackwood B. Factors that impact on the use of mechanical ventilation weaning protocols in critically ill adults and children: a qualitative evidence-synthesis. Cochrane Database Syst Rev. 2016;2016(10):CD011812. doi: 10.1002/14651858.CD011812.pub2 27699783 PMC6458040

[14] O’Brien BC, Harris IB, Beckman TJ, Reed DA, Cook DA. Standards for reporting qualitative research: a synthesis of recommendations. Acad Med. 2014;89(9):1245–51. doi: 10.1097/ACM.0000000000000388 24979285

[15] Braun V, Clarke V. Using thematic analysis in psychology. Qual Res Psych. 2006;3:77–101.

[16] Guest G, Namey E, Taylor J, Eley N, McKenna K. Comparing focus groups and individual interviews: findings from a randomized study. Int J Soc Res Methodol. 2017;20:693–708.

[17] Lambert SD, Loiselle CG. Combining individual interviews and focus groups to enhance data richness. J Adv Nurs. 2008;62(2):228–37. doi: 10.1111/j.1365-2648.2007.04559.x 18394035

[18] Silverman D. Interpreting Qualitative Data: Methods for Analysing Talk, Text and Interaction. 5th ed. London: SAGE Publications Ltd; 2015.

[19] Blackwood B, Morris KP, Jordan J, McIlmurray L, Agus A, Boyle R, et al. Co-ordinated multidisciplinary intervention to reduce time to successful extubation for children on mechanical ventilation: the SANDWICH cluster stepped-wedge RCT. Health Technol Assess. 2022;26(18):1–114. doi: 10.3310/TCFX3817 35289741

[20] Bloos F, Muller S, Harz A, Gugel M, Geil D, Egerland K, et al. Effects of staff training on the care of mechanically ventilated patients: a prospective cohort study. Br J Anaesth. 2009;103(2):232–7. doi: 10.1093/bja/aep114 19457893

[21] Castellanos I, Martin M, Kraus S, Burkle T, Prokosch HU, Schuttler J, et al. Effects of staff training and electronic event monitoring on long-term adherence to lung-protective ventilation recommendations. J Crit Care. 2018;43:13–20. doi: 10.1016/j.jcrc.2017.06.027 28826081

[22] Davis KF, Napolitano N, Li S, Buffman H, Rehder K, Pinto M, et al. Promoters and Barriers to Implementation of Tracheal Intubation Airway Safety Bundle: A Mixed-Method Analysis. Pediatr Crit Care Med. 2017;18(10):965–72. doi: 10.1097/PCC.0000000000001251 28654550 PMC5628113

[23] Larsen MH, Johannessen GI, Heggdal K. Nursing interventions to cover patients’ basic needs in the intensive care context—A systematic review. Nurs Open. 2022;9(1):122–39. doi: 10.1002/nop2.1110 34729954 PMC8685812

[24] Trogrlic Z, van der Jagt M, van Achterberg T, Ponssen H, Schoonderbeek J, Schreiner F, et al. Prospective multicentre multifaceted before-after implementation study of ICU delirium guidelines: a process evaluation. BMJ Open Qual. 2020;9(3). doi: 10.1136/bmjoq-2019-000871 32948600 PMC7511605

[25] McCall M, Cahill N, Murch L, Sinuff T, Bray T, Tanguay T, et al. Lessons Learned From Implementing a Novel Feeding Protocol. Nutrition in Clinical Practice. 2014;29:510–517. 24757062 10.1177/0884533614531047

[26] Rosa RG, Teixeira C, Sjoding M. Novel approaches to facilitate the implementation of guidelines in the ICU. J Crit Care. 2020;60:1–5. doi: 10.1016/j.jcrc.2020.07.014 32731099

[27] Wilson J, Tracey E, Ponnala J, Rodriguez-Hobbs J, Crowe T. An ICU Expansion of a Novel Chaplain Intervention, "This is My Story," to Support Interdisciplinary Medical Teams Delivering Care to Non-Communicative Patients in an Academic Medical Center. J Relig Health. 2023;62(1):83–97. doi: 10.1007/s10943-022-01567-9 35482270 PMC9047461

[28] Hawe P. Lessons from complex interventions to improve health. Annu Rev Public Health. 2015;36:307–23. doi: 10.1146/annurev-publhealth-031912-114421 25581153

[29] Hawe P, Shiell A, Riley T. Complex interventions: how "out of control" can a randomised controlled trial be? BMJ. 2004;328(7455):1561–3. doi: 10.1136/bmj.328.7455.1561 15217878 PMC437159

[30] Gesell SB, Prvu Bettger J, Lawrence RH, Li J, Hoffman J, Lutz BJ, et al. Implementation of Complex Interventions: Lessons Learned From the Patient-Centered Outcomes Research Institute Transitional Care Portfolio. Med Care. 2021;59(Suppl 4):S344–S54. doi: 10.1097/MLR.0000000000001591 34228016 PMC8263141

[31] Baker R, Camosso-Stefinovic J, Gillies C, Shaw EJ, Cheater F, Flottorp S, et al. Tailored interventions to address determinants of practice. Cochrane Database Syst Rev. 2015;2015(4):CD005470. doi: 10.1002/14651858.CD005470.pub3 25923419 PMC7271646

[32] Collinsworth AW, Priest EL, Campbell CR, Vasilevskis EE, Masica AL. A Review of Multifaceted Care Approaches for the Prevention and Mitigation of Delirium in Intensive Care Units. J Intensive Care Med. 2016;31(2):127–41. doi: 10.1177/0885066614553925 25348864 PMC4411205

[33] Ersson A, Beckman A, Jarl J, Borell J. Effects of a multifaceted intervention QI program to improve ICU performance. BMC Health Serv Res. 2018;18(1):838. doi: 10.1186/s12913-018-3648-y 30404646 PMC6223055

[34] Kamdar BB, Yang J, King LM, Neufeld KJ, Bienvenu OJ, Rowden AM, et al. Developing, implementing, and evaluating a multifaceted quality improvement intervention to promote sleep in an ICU. Am J Med Qual. 2014;29(6):546–54. doi: 10.1177/1062860613509684 24270169 PMC4141028

[35] Ervin JN, Kahn JM, Cohen TR, Weingart LR. Teamwork in the intensive care unit. Am Psychol. 2018;73(4):468–77. doi: 10.1037/amp0000247 29792461 PMC6662208

[36] Stocker M, Pilgrim SB, Burmester M, Allen ML, Gijselaers WH. Interprofessional team management in pediatric critical care: some challenges and possible solutions. Journal of Multidisciplinary Healthcare 2016;9:47–58. doi: 10.2147/JMDH.S76773 26955279 PMC4772711

[37] Donovan AL, Aldrich JM, Gross AK, Barchas DM, Thornton KC, Schell-Chaple HM, et al. Interprofessional Care and Teamwork in the ICU. Crit Care Med. 2018;46(6):980–90. doi: 10.1097/CCM.0000000000003067 29521716

[38] Pronovost P, Needham D, Berenholtz S, Sinopoli D, Chu H, Cosgrove S, et al. An intervention to decrease catheter-related bloodstream infections in the ICU. N Engl J Med. 2006;355(26):2725–32. doi: 10.1056/NEJMoa061115 17192537

[39] Wheelan SA, Burchill CN, Tilin F. The link between teamwork and patients’ outcomes in intensive care units. Am J Crit Care. 2003;12(6):527–34. 14619358

[40] Balit CR, LaRosa JM, Ong JSM, Kudchadkar SR. Sedation protocols in the pediatric intensive care unit: fact or fiction? Transl Pediatr. 2021;10(10):2814–24. doi: 10.21037/tp-20-328 34765503 PMC8578750

[41] Hellyer TP, McAuley DF, Walsh TS, Anderson N, Conway Morris A, Singh S, et al. Biomarker-guided antibiotic stewardship in suspected ventilator-associated pneumonia (VAPrapid2): a randomised controlled trial and process evaluation. Lancet Respir Med. 2020;8(2):182–91. doi: 10.1016/S2213-2600(19)30367-4 31810865 PMC7599318

[42] Kucher NM, D DS, Fischer GA, Davey CS, Gupta S. Implementation of a Critical Asthma Protocol in a Pediatric ICU. Respir Care. 2021;66(4):635–43. doi: 10.4187/respcare.07944 33504572 PMC9993982

[43] Miller AG, Haynes KE, Gates RM, Zimmerman KO, Heath TS, Bartlett KW, et al. A Respiratory Therapist-Driven Asthma Pathway Reduced Hospital Length of Stay in the Pediatric Intensive Care Unit. Respir Care. 2019;64(11):1325–32. doi: 10.4187/respcare.06626 31088987 PMC6945301

[44] Mouncey PR, Wade D, Richards-Belle A, Sadique Z, Wulff J, Grieve R, et al. A nurse-led, preventive, psychological intervention to reduce PTSD symptom severity in critically ill patients: the POPPI feasibility study and cluster RCT. Health Technol Assess. 2019;7(30). 31465162

[45] Pronovost PJ, Murphy DJ, Needham DM. The science of translating research into practice in intensive care. Am J Respir Crit Care Med. 2010;182(12):1463–4. doi: 10.1164/rccm.201008-1255ED 21159904

[46] Schreiber AF, Ceriana P, Ambrosino N, Malovini A, Nava S. Physiotherapy and Weaning From Prolonged Mechanical Ventilation. Respiratory Care. 2019;64(1):17–25. doi: 10.4187/respcare.06280 30206129

[47] Bacci SLL dos S, Pereira JM, Chagas AC da S, Carvalho LR, Azevedo VMG de O. Role of physical therapists in the weaning and extubation procedures of pediatric and neonatal intensive care units: a survey. Brazilian Journal of Physical Therapy. 2019;23(4): 317–23. doi: 10.1016/j.bjpt.2018.08.012 30249437 PMC6630111

[48] Woo BFY, Lee JXY, Tam WWS. The impact of the advanced practice nursing role on quality of care, clinical outcomes, patient satisfaction, and cost in the emergency and critical care settings: a systematic review. Human Resources for Health. 2018;15(1):1–22. 10.1186/s12960-017-0237-9 PMC5594520 28893270

[49] Grant A, Treweek S, Dreischulte T, Foy R, Guthrie B. Process evaluations for cluster-randomised trials of complex interventions: a proposed framework for design and reporting. Trials. 2013;14:15. doi: 10.1186/1745-6215-14-15 23311722 PMC3600672

[50] McIntyre SA, Francis JJ, Gould NJ, Lorencatto F. The use of theory in process evaluations conducted alongside randomized trials of implementation interventions: A systematic review. Translational Behavioural Medicine. 2020;10:168–178. doi: 10.1093/tbm/iby110 30476259

