# Supplementary material for: Delivery of a novel intervention to facilitate liberation from mechanical ventilation in paediatric intensive care: A process evaluation
Source: PLoS One. 2023 Nov 27;18(11):e0293063. doi: 10.1371/journal.pone.0293063 (PMC10681213; doi:10.1371/journal.pone.0293063)
Supplement: S1 Appendix — (DOCX) [file pone.0293063.s001.docx]

**Additional file 1: SANDWICH implementation**

| **Strategy** | **Activities** |
| --- | --- |
| Establishment of SANDWICH trial team to oversee all aspects of trial delivery | Provision of clear lines of responsibility, routes of communication and support of PICU research teams. Availability of:   - Chief Investigator (CI) - Trial Implementation Manager - Clinical Trial Unit Team - Process Evaluation Lead |
| Paediatric Intensive Care Unit (PICU) appointed trial teams to maximise local-level engagement in trial | 1. Participating PICUs nominated at least one SANDWICH research nurse with responsibility for:   - liaising with the trial team, primarily the implementation manager - training clinical staff on the intervention during an 8-week training - collecting trial data in the usual care and intervention periods   Payment for the SANDWICH nurses’ time was reimbursed to the hospital from the trial grant.  2. PICU research nurses identified multidisciplinary SANDWICH trainers, responsible for planning, organising and rolling out intervention training to all clinical staff during an eight-week training period. |
| Multi-faceted education and training on intervention for all clinical staff involved in patient care to maximise understanding of and engagement in trial | 1. Trial implementation manager responsible for:   - - helping to develop education and training materials and resources; - - visiting all PICUs to train local PICU trainers.   2. A purposively developed SANDWICH course delivered training and assessment of staff understanding of intervention components and underpinning clinical evidence supporting interventionised weaning. Course delivered online using LearnPro  (https://learnpro.co.uk/products/lms/learning-management-system). All clinical staff involved in the delivery of the intervention required to successfully complete the online training and assessment. Target for achieving staff training was 80% within the 8-week training period. Course compliance measures fed back to individual PICU trainers during the training period for monitoring purposes.  3. Online course supplemented by a Trainer’s Manual with power point slides, video and online and written educational material providing detail around the components of the intervention. |
| Ongoing support and feedback for PICUs to maximise engagement in trial | 1. Trial implementation manager provided ongoing, targeted implementation support and guidance for each participating PICU as required.    2. Monthly online site investigator meetings between PICU research teams and SANDWICH trial team (CI, trial implementation manager, PE lead).  3. Additional resources produced to provide reminders and facilitate adoption of the intervention - included flyers, posters, screensavers, lanyards, badges and banner message pens.  4. Feedback on PICU intervention adherence proportions on three occasions during the trial to the SANDWICH nurse to disseminate within the PICU and promote adherence. Feedback included the unit’s own adherence values alongside an anonymised unit’s high adherence values for comparison. |
| PICU trial advocacy and support to maximise understanding and delivery of trial. | PICU appointed SANDWICH multi-disciplinary ‘champions’ to promote implementation, assist with training queries and provide local support when necessary. |
