# Supplementary material for: Delivery of a novel intervention to facilitate liberation from mechanical ventilation in paediatric intensive care: A process evaluation
Source: PLoS One. 2023 Nov 27;18(11):e0293063. doi: 10.1371/journal.pone.0293063 (PMC10681213; doi:10.1371/journal.pone.0293063)
Supplement: S3 Appendix — (DOCX) [file pone.0293063.s003.docx]

**Additional file 3: Process of theme development (following Braun & Clarke, 2006) [16]**

| **Phase** | **Activity** |
| --- | --- |
| Familiarisation | Repeated reading of transcripts enabled detailed familiarisation with their content. |
| Generating initial codes | Based on this reading, specific portions of text were assigned a code, which reflected “semantic content”, that is, the explicit / overt meaning of participants’ responses. |
| Search for themes | The relationship (similarities and differences) between these initial codes was considered during subsequent rounds of analysis, during which codes could be lost, amended or new ones created as their content and meaning was compared in relation to one another and to the dataset in its entirety. This extended process brought different codes together to form a “candidate” thematic framework. |
| Reviewing themes | The candidate framework was refined, involving a moving back and forth between the codes and the themes in which they were embedded and the themes themselves. At this stage, embryonic themes were amended as a final set was developed. A concluding re-reading of the entire dataset ensured that these themes adequately accounted for all data. |
| Defining and naming themes | Each of the identified themes was appropriately labelled and explained. Labels reflected the essential meaning and were the basis of a narrative, which made explicit what the theme addressed / captured, including through the use of relevant quotes. |
| Producing the report | The analysis acted as the basis of empirically informed arguments concerning the processes involved in trial delivery and their implications for trial outcomes. |
