# Supplementary material for: Delivery of a novel intervention to facilitate liberation from mechanical ventilation in paediatric intensive care: A process evaluation
Source: PLoS One. 2023 Nov 27;18(11):e0293063. doi: 10.1371/journal.pone.0293063 (PMC10681213; doi:10.1371/journal.pone.0293063)
Supplement: S4 Appendix — (DOCX) [file pone.0293063.s004.docx]

**Additional file 4: Supporting quotes**

| **Theme 1. Training promotes understanding and skill set**  *“I think it was good. I remember e-learning, it was quite difficult to get done here, the practicalities of that, I think because a lot of it had sound to it all, video components, to do that at work was difficult. Quite a few people ended up having to do that in their own time, some of us had to do it anyway. But other than that, I don’t remember it being like bad or a challenge or anything like that.”* (PICU Bedside Nurse, S10)  *“But again, it’s been a fantastic experience for all of us, the results have been exceptionally good for us, the training has been remarkable, there’s a wealth of knowledge out there and I see it to be part of the ICU’s future.”* (PICU Ward Manager, S16)  *“So the training package was really good and, as far as I’m aware from looking at how we did… that worked well.”* (PI, S16)  *“I do think it was done really well.”*  *“Yes, really thorough.”* (PICU Senior Nurses, S18)  *“I think there was some really good things about it being implemented. I think the training was very good. We’re a big unit, as you know, and have a particularly large cohort of nurses and the training that went on was very good.”* (PICU Physio, S03)  *“… the questions and that was quite full on. A lot of work and a lot of the pre-questions.* … *although it was long, it was all necessary, because it was a completely new process and it was not similar to anything that we were doing. So I think if you do not have that level of training then it wouldn’t work. So although it was long, it was probably necessary.”* (PICU Senior Nurse, S03)  *“… initially I was on annual leave or I was away…but then afterwards I did, from one of the girls [research team nurse], like a bedside sort of training… I found the bedside training there in the environment a lot more useful…it was like you’re doing it for a patient in your bed space, just like a learning module.”* (PICU Bedside Nurse, S03)  *“I remember doing the online training…but I did think there must be a quicker way of doing this. It’s like there’s only two or three core messages, I suppose it's different for different people maybe but I remember thinking at the time, you know, we really support these trials but we should try and make them as easy as possible to actually … So maybe I’m being a bit arrogant in saying half of it we could have done without. There isn’t an easy solution. There were certainly bits that I found useful and bits that I was just like oh God, this is painful.”*  *“…I think there was quite a lot of frontloading of teaching and training in the ward. It was actually good that such a, I wouldn’t say complex intervention, but an intervention with multiple parts to it… There was quite a lot of frontloading, there was quite a lot of video tutorials, handheld presence on the shop floor before it was being rolled out in that phase where we were being taught the intervention before we applied it and changes made to the handover, appropriate paperwork, sign posting.”* (PICU Consultants, S03)  *“…I was trained by one of the consultants, and it was really good and, yes, it did give you a good idea when you were gauging when a patient was nearly ready for extubation … speaking for me, because I literally have just started, so it probably was good in that sense for me … “So it was like an individual teaching session, things were explained to you, and I do think it was adequately explained and if you'd any questions or anything you could bring it to the nurse or speak to the consultant, who was also very good and very hands on.”* (PICU Bedside Nurse, S02)  *“I think, like with everything, it’s very hard to make it specific for certain people, and there were bits of it that I didn’t need but I understood why it was as it was, because without going into everybody’s background how do you know what they do and don’t understand.”* (PICU Consultant, S02)  *“I didn’t find it too bad, personally. I know a lot of people were saying it was long winded but I didn’t find it too bad.”* (PICU Bedside Nurse, S17)  *“… if we didn’t have online training or just some sort of theory to back it up or underlie it then it might have taken a lot longer for us to get our heads around it.”* (PICU Bedside Nurse, S15)  *“… and so when they [unit staff] wanted to do it, they all did it, and when they found the module was really good, it was even better…”* (PI, S15)  *“I think it was fine, bedside, and it was fine to go through, I didn’t get to anything "oh, I’ve struggled," so they must have done a decent job … definitely face to face was far more successful in terms of how people understood the process.”*  *“I did think it was useful, but I think it was more beneficial just once we’d just started doing it with people. It did take people a while I think, to get into it, but yes, it was useful to have a background of why we were doing it and understand the basics.”* (PICU Bedside Nurses, S13)  *“It was quick snapshot of what it actually was. Rather than going into all of the background and loads of information that I didn’t need to know, she just did a really quick overview snapshot of it, and that made sense to me.”*  *“I found the online bit most beneficial. The face to face training, I came away from that with no more understanding than what I did beforehand.”* (PICU Senior Nurses, S04)  *“Not particularly useful or necessary [online training] … I think probably perhaps just from a very personal point of view, I’m more of a doer. So I have to keep doing it to understand it.”* (PICU Bedside Nurse, S04)  *“I think it would be easier if it was just one training package, if it wasn’t several modules that they could just sit and do all in one go and that was it…”* (N/PICU Research Nurse, S08)  *“The bedside sit-down and go-around training was good, wasn’t it. I think the one-to-one training was more effective than the series of videos and stuff, is what I think. I don’t think the online training…really helped”* (PICU Consultant, S11)  *“It was very useful”* (PICU Registrar, S06) |
| --- |
| **Theme 2. Ease and flexibility of use**  **Sub-theme: Intervention acceptability**  *“I think it definitely worked, yes. There was nothing really that I thought oh, we need to include this on there. I think all the bases were covered.”*  *“I felt like I could almost do it during ward round if ward round started early…I felt it was quick enough for me to do it whilst ward round…”*  *“Yes, me too…Because it was part of the bundle, you just made it as part of the bundle and it was a lot easier to access and sort out … To me, it was just part of the nursing assessment, I’d just do it first thing in the morning.”* (PICU Bedside Nurses, S03)  *“I think, because it was quite useful, you didn’t feel…you do get that thing sometimes in the bed space, “oh god it’s another bit of paperwork”, but it’s so quick to do it doesn’t actually really take up that much time.* … *I found the whole system worked for the nurses, went really, really well in the bed space, because you’ve got your comfort score, you’ve got everything on a piece of paper in front of you, you’re being prompted to do what you need to do when you need to do it…”* (PICU Bedside Nurse, S03)  *“So in terms of the bundle, it was a lot of information but I think it was handled well… and the way that it was presented was really helpful, the colours and the tables and just the way that the bundle, it just made it easy to use, very user friendly.”* (PICU Senior Nurse, S03)  *“It was very, very easy to do, very easy, less than 30 seconds really just to go tick, yes, that, or yes, that.”* (PICU Senior Nurse, S05)  *“I think, on the whole, they were done. It was easy, really easy.”* (PICU Research Nurse, S07)  *“I think it’s quite straightforward and I think it was good to look and think, oh actually, you know, they have got a good cough, their oxygen is below 45%, it was quite quick and simple to do.”* (PICU Bedside Nurse, S07)  *“It was more paperwork, it was another thing to remember… you’ve done an SBT and comfort score is saying they’re comfortable, that you can extubate them… to then… I know you could do it reflectively, but to sit there and then have to do the paperwork...”*  *“I just built it into my day, so I didn’t find that it was time consuming or anything like that. From that point of view, I found it quick and easy to do, I guess. I think from my kind of view…I was doing it like a tick-box exercise and I kind of knew the outcome before it gave me the score, if that make sense.”* (PICU Bedside Nurses, S07)  *“I think the checklist was easy to understand.”* (PICU Bedside Nurse, S17)  *“Yes, I thought that was good, because you meet the criteria…So even as a more junior member of staff…they can fill out the checklist…because it's numbers, and then they can say “oh, they do meet the criteria”, and then you can speak to…so I think that kind of made it easier because it gives you a number and a definite rather than a subjective.”* (PICU Bedside Nurse, S06)  *“So I think it was good… I think the paperwork was easy. So you’d easily know which child could be easily identified and which child you were to do it on. So it was all very straightforward.”* (PICU Senior Nurse, S06)  *“It doesn’t take very long…it's just a quick tick box. So I don’t think it was a burden and I think it did help you think, okay, well can I extubate and, if so, I need to raise this as a… So yeah, I think it was good.”* (CICU Bedside Nurse, S09)  *“…because it wasn’t…you’re not doing it every hour. It was once or twice a shift, and that was perfectly manageable, I thought.”* (PICU Bedside Nurse, S10)  *“So it was all kind of a lot of change at the same time. And when it was utilised, it was great, people really got on board with the checklists, they were doing them every ward round and everything, which made people talk about comfort scores, which we’d never really done before, which was good. But I guess the main problem continuously is documentation. So documenting these additional bits, so, documenting that they’ve given a leaflet, documenting that they’ve done the ward round checklist, documenting when they’ve done a screen or an SBT.”* (PICU Research Nurse, S10)  *“It was easy enough once you kind of knew what you were doing. At the beginning everyone had sort of different ideas as to how it worked and where to write things.”* (PICU Bedside Nurse, S15)  *“To be honest, none of it took very long, it wasn’t a huge workload… “I mean, it doesn’t take more than ten minutes in the whole shift. So to me, it didn’t really add much, it was pretty straightforward; yes, yes, no, no, one, two three, that's it.”* (PI Senior Nurse, S15)  *“… it couldn’t have been easier, to be fair, something as complicated as this. This is a very…so much variability. I think it definitely was easy to follow and not very prescriptive … the flexibility was really good and that helped the buy-in and that helped embedding in practice.”*  *“… some of our colleagues didn’t like putting children under pressure support … but when this came in and they looked at it as that it is being reviewed by many experienced intensivists, they did buy into it and there wasn't any resistance.”* (PI Consultants, S15)  *“It’s quite simple and quite quicker, I think”.*  *“It’s just like doing a GCS, you just do it, don’t you.”*  *“I think at first it was…because our comfort score sheet before was just one sheet and it was very similar to the comfort score sheet for the Sandwich stuff, I don’t know if you felt the same way, it seemed to seem more complicated than actually it was, and once you get into it it was absolutely not an issue at all to do.”*  *“It’s just like anything, isn’t it, just like changing anything, it takes a little while to get used to, but it was never complicated.”* (PICU Bedside Nurses, S18)  *“I think if you hadn’t been flexible you wouldn’t have gotten so many people on board, so many units on board. We were particularly reassured by the fact that when you said, "okay, if you already planned to introduce your nurse weaning, whatever, it’s okay, continue, we won’t stop you doing that," and actually what happened was we moved forwards faster with Sandwich than we did with our own plans, and I think that flexibility helped.”* (PI, S18)  *“I mean, the places where the SBT numbers don’t work as well are in people with chronic lung problems weaning off onto non-invasive ventilation, so obviously the SBT numbers, the numbers that we’re never aspiring to have, even when they’re off a ventilator, but those who adapted fairly effortlessly as far as it goes, so we’ve taken that on board, we adapt more appropriate pressures rather than going down to the known ones on the SBT trial. So that hasn’t caused a problem.”* (PICU Consultant, S18)  *“So that five on five, I can see from a research aspect why it’s important, but implementing it on a clinical practice point of view, it’s a bit more difficult, you need to have some sort of leeway either side… But I think, overall, the protocol was completely appropriate and it was workable and feasible in terms of daily practice; I didn’t find it too much of a hindrance.”* (PICU Consultant, S04)  *“… but we obviously knew to just use our own judgement as well. But there was a nurse section to be able to do that, to say whether, if they’d scored low, that they were over sedated, but we had a reason why and we could write that. So I liked that part of it.”* (PICU Bedside Nurse, S13)  **Sub-theme: Stasis in the absence of explicit direction**  *“It wasn’t pointless necessarily, but we didn't do anything with it either. We had to write a lot to explain one of the three numbers you’d chosen, and then no one would ever look at it again.”* (PICU Bedside Nurse, S15)  *“I don’t think it’s very individual. There seems to be a genuine acceptance of between 12 and 17.”* (PICU Senior Nurse, S06)  *“But I do agree with the sedation part, because I think everything else was followed quite well but the sedation, the comfort score aim wasn’t really discussed. I guess it was kind of assumed that most people would want it 12 to 17, but that was the one thing that I did notice was touched on as much as everything else on the ward rounds.”*  *“It’s quite a big jump, I think…Sometimes on 12, you can be quite flat on that, or you might have a patient who's on 15 who could be really wild or they could be settled.”*  *“…because there’s a big difference between a 12 and 17, and I don’t think I’d want a child at 17 but equally, I think it could have been like 15ish, like 14/15 that sort of area. 12 often actually is pretty well sedated…”* (PICU Bedside Nurses, S10)  *“The targets were set…so that was good, but the range was quite a lot, I'd say… If you score 17 they’re actually quite awake, compared to 12. So it’s completely different.”* (PICU Senior Nurse, S16)  *“… but the range was quite a lot, I'd say…If you score 17 they’re actually quite awake, compared to 12. So it’s completely different.”* (PICU Bedside Nurse, S16)  *“The things I didn’t like about it is the whole sedation score. I know it was a kind of light sedation with weaning and extubation…It was said in handover and the ward round "what’s the colour?" "Oh something, green..." "That's fine, that's fine."…I think the nurses will tell you if they’re not happy about sedation, regardless of the study or not. So from the idea of talking about sedation, there’s that many things to cover, if you’re happy with sedation it's not one of the things you bring up. So from that perspective, I didn’t think that was particularly helpful, trying to score and trying to work out where they were on the sedation spectrum.”*  *“…I think that’s [the 2 hour window] fine for the patient. I think it's difficult for the unit, if that makes sense…I like the idea you've got two hours, if I'm ready to get them off, that’s fine. It's quite common when you're doing a ward round in the morning to say "I want to extubate here, here and here", and then by the afternoon you’re like "I haven’t done [the extubation] because I’ve been too busy here. I feel like that was the difficulty, trying to keep to that two-hour window.”* (PICU Consultants, S01)  *“… and so it became a sort of a pointless exercise where they would just say "everyone’s green," or the feeling would be they should be green.”* (PICU Senior Nurse, S01)  *“…there quite a few… If I’m being really honest, there are a lot of patients that, according to Sandwich, would have met the criteria for SBT but we justified not doing it because of other worries…. if you put them on SBT and then two hours later you’ve got to make a decision, that’s actually a soft resource implication of you’re going to have to put them on the SBT, then you’ve going to have to go and look at them a little while later and a little while later again… whereas if there was someone dedicated to doing that, I actually think we might have surprised ourselves a bit more often…”* (PI, S01)  *“There was a lot of patients who were on it [SBT] for longer, because they were put on and no one go to back to them. So after ward round the doctor would come around, put you onto CPAP, so you’ve made that, kind of, 5 a.m., they were reviewed, they were put onto CPAP, great, ready for ward round, but then ward round would happen and then, because you’ve got the ECMO patient and the head injury and…such and such, people still weren’t getting to the bedside…we would have extubated patients sooner, but quite often they were sat for five, six hours…”* (PICU Research Nurse, S01)  *“It was confusing in the beginning. I remember that being very confusing in the beginning, because we weren’t sure what you do at two hours… I think it was individual, it depends on the patient… if you got to the end of two hours then you’d put them on less or maybe just a tiny little bit of support or something just so they didn't get tired. So it was patient… individually assessed.”* (PICU Bedside Nurse, S13)  *“… it [ the Comfort Score] wasn’t always discussed though… It was 12 to 17, unless otherwise stated.”* (PICU Bedside Nurse, S02)  *“… if you get a child breathing through a narrow tube for more than half an hour or so you start beginning to get atelectasis. So I preferred not to wait for the two hours. You know, if they’ve done 15 minutes, good, if they’ve done 30 minutes, that’s it, done. I prefer not to leave it for two hours…. And in fact, sometimes, what could happen is that a child who was ready to extubate, they've been breathing through a narrow straw for two hours, they’ve lost some lung volume and then they’re no longer extubatable.”* (PI, S17)  *“The slightly, not negative, but things that I thought might be indifferent is that sometimes it becomes an automatic response. For example, comfort green and saturations above X is a default setting, especially when you’re busy, and sometimes it's not necessarily always given the in-depth thought process. I'm not saying it’s right or wrong…”* (PICU Consultant, S04)  *“But nobody says their comfort score should be this. You just go through and there’s a medical plan on our notes and it just says comfort score green, 12 to 17, pretty much what we always see.”* (PICU Bedside Nurse, S04)  *“So especially from a physio point of view I was like ah! And the other thing alongside of that was some people would just leave, I thought, children on it for too long, on the SBT, where actually then sometimes they were fatiguing, and I think actually they could have just done it for a shorter time, reassessed and then extubate, that may have been better.”* (PICU Physio, S04)  *“So two hours probably is…two hours allows a lot of other things to happen, rather than people focusing on "I’m doing an SBT, I’m going to watch this for a while and see what happens."”* (PICU Consultant, S04)  *“The one confusion I see I think was particularly going back to sedation was that I came across a few cases where the child…may have been in the green zone but was still sedated, and then nurses were doing SBTs and then saying that they’d failed the SBT, but actually I wouldn’t have actually done an SBT on the amount of sedation the child was on. And then it was quite frustrating...”* (PICU Consultant, S07)  *“I think that you do the Spontaneous Breathing Trial and they met criteria at 30 minutes, they kind of passed, but I think we had lots of kids that were on for far more than the 30 minutes and they weren’t extubated at two hours to say they’ve passed; they’re ready to extubate. I think it was an acceptance of okay, we can extubate this child, we’ll do it whenever we’re ready to do it, as opposed to actually they’ve passed the 30 minutes and we should try and take the tube out by two hours or within the next few hours. That wasn’t the case. It was “we know they’ve met it; we know we can take that tube out; we’ll take the tube out when it suits us. … I didn’t feel like the conclusion of Sandwich was the tube coming out or sedation coming off. It was to try and reduce that, and for me, I didn’t get the ownership of once they’ve hit that Spontaneous Breathing Trial we should take that tube out quicker.”* (PICU Physio, S03)  *“But there were some occasions that I observed where there was no closed loop feedback back to the medic or the ANP to say “you know we’ve been SBT five on five for the last two hours, why are we not extubating this child?” So it's… like “oh, I forgot”, if you got distracted. I really feel if there was an emergency something else, that I appreciate, but there were also occasions where there were no emergencies but there was this failure of closed loop communication, if that makes sense.”* (PI, S03) |
| **Theme 3: Promotes proactivity in weaning**  **Sub-theme: Effectiveness of ward round planning**  *“I think when the doctors started asking for it [sedation status] on ward round… that was the big thing. I think the nurses before were writing it and they were writing the number down and nobody actual cared what this number is for. I think for doing it for the study and the doctors being involved and everyone discussing it basically gave it a sense of worth and actually the nurses then bought in, "oh yes, I need to do this, they’re going to ask me, I’ve got to do this."* (PICU Senior Nurse, S11)  *“… the checklist on the ward round is actually quite hard to get done every day…. People just forgot to do it. It didn’t get done every day. We tried giving it to different people in the teams to try and take ownership of it… they will sit with this at the back and they wouldn’t pipe up… The knock-on effect was that we didn’t always talk about those things on the checklist…”* (PICU Consultant, S11)  *“I think they always asked, but definitely, again, they would always ask [in ward rounds] “what comfort scores are you on? What are you aiming for? What can we do to get to your…”* (CICU Bedside Nurse, S09)  *“… and like comfort scores weren’t regularly mentioned. There were some consultants who were like obviously really on board with it and every ward round would say it, but it was also kind of like an afterthought at the end [for others], as everybody would be walking off then they’d just kind of go "aiming for a comfort score of. … “You always know when you’ve got a patient, if they’re planning on extubating… Because they [consultants] do mention it and they’ll say like "we’re hoping to extubate later today, so we need to wean sedation…"*  *“I think it’s definitely a valid component [Comfort Scoring] and important to ask, and I think, again, it varies on who is a consultant for that day as to whether they’d…but when it comes to a set plan that's clear to everyone, that doesn’t happen that regularly, I don’t think.”* (N/PICU Bedside Nurses, S09)    *“I think where it fell down in the process was the ward round and how often it actually was discussed or see people filling in the sheet. We filled in some of the sheets and the nurses did, we never saw a doctor filling in the sheets. Sometimes there wasn’t a sheet, their file didn’t go in, that kind of thing. So I there it almost seemed to feel like it was the last thing to think about if everything else was busy…”* (PICU Physio, S18)  *“I wouldn’t say all the time… But sometimes. I think it was consultant dependent. Some were more receptive and more involved in it and would set the targets and say this is where we want them to be, we want to try an SBT in the morning, that sort of thing, and then others, it wasn’t mentioned unless we brought it up.”*  *“They did go around each bed space with the doctors and I think it was added as like a star in their doctors handovers, so they had to ask, and they did, yes, and if some forgot then we would ask what score we were aiming for. I think some people, without asking the doctors, knew what score we were going… we just kind of got to know actually probably what we were scoring for but the doctors did tell us every morning.”* (PICU Bedside Nurses, S13)  *“So the ward round checklist, documenting whether or not we’d done that, we often forgot to do… it depends which consultant. So [name of PI] who is obviously very for Sandwich was very good at it, others were not as…To be honest, it's probably a combination between the doctors and the nurse in charge, because our nurse in charge would have the checklist, so if they were the one holding it and they were saying well actually before we move on let’s do the checklist, then it would happen. If the checklist got forgotten about or whatever else then it probably wasn’t so likely to happen. It was often the nurse instigating it for the consultant to do.”* (PICU Bedside Nurse, S10)  *“… I think because we introduced a new checklist at the same time and it was a lot more all-encompassing, so we kind of did it so that it wouldn’t be another thing that we rammed down people’s throats, so it kind of encompassed everything, but it was definitely about talking about SBTs, talking about screens, talking about comfort scores, it was all included in that…So ward round definitely improved when it was done, but again it’s one of those things, unless the nurse in charge was really proactive and they’re like "where’s the checklist?" then sometimes it was missed. I kind of felt that medics were reluctant to pick a comfort score as well…”* (PICU Research Nurse, S10)  *“There are certain nurses in charge who will make sure that they chase it up and they will be like a dog with a bone and will go over it every day, and there’s others that won’t, and when those that do press it, press it, most of the consultants, 90% of the time, are very receptive to it and thankful for it, but if it’s not pressed then it’s just exhausting that it always comes down to the nurse, that if the nurse is on it, it gets done, and if the nurse isn’t on it then it doesn’t… And you have to kind of jump in at the beginning and say "right, let’s do the checklist." To be honest, you go through everything and then somebody says "oh let’s do the checklist," because at that point everybody feels like they know what the plan for the day is and you feel like you’re rehashing old information, just being on top of it.”*  *“The ward round checklist, when it worked, it worked really well, and that was the part that was missing all these years, but that was the part that was probably the hardest to actually implement because there wasn’t consistency…but I don’t feel like that that was mirrored from a consultant point of view… [i]n their ownership... because if they didn’t properly own it then the registrars didn’t get on board. So you might only have one or two consultants who were rigidly sticking to the "right, let’s do the ward round checklist," because I mean we’ve battled for years to do some kind of efficient ward round, kind of like following a mnemonic or some kind of, you know. So where it worked, it worked very well…”* (PICU Senior Nurses, S10)  *“And especially on ward round… Sandwich and comfort was always mentioned, it always came up and you always had to report back on it. What’s your SBT and what’s the comfort scores and what about Sandwich and the documentation was something that was paid close attention to, it was something that was religiously done.”* (PICU Bedside Nurse, S02)  *“…and they’d come around at handover and colour code [for sedation management] your patients, that didn’t always happen, it depended how busy it was and it depended who was the team leader, but it was useful when it did happen.”* (PICU Bedside Nurse, S01)    *“…to be honest with you, I don’t think it was completely well managed at times. I just wonder if it’s like anything, it was just such a big change to what we do that the biggest thing was reminding people at the handover to actually discuss Sandwich, and the poor physio was quite often shouting "Sandwich" from the corner when we were moving onto the next patient.”* (PICU Senior Nurse, S01)  *“It’s been a little bit challenging to remind people that we need to set the target on a daily basis for every child who is ventilated invasively, but I think we have been getting better with the months.”* (PICU Consultant, S05)  *“I think we did. I think in ward rounds for me it took the sticker sometimes to remind us. You would have ward rounds where you might have such a lot going on about a particular child and you’ve got that time pressure of getting around the patients and so I needed the reminder. … but I think it was a … process we saw develop over the trial.”* (PICU Consultant, S03)  *“… or they’d done it [Comfort Scores] but it hadn’t been discussed on ward rounds so then it hadn’t quite moved on.”* (PICU Physio, S03)  *“So that side of things, in ward round, is trying to remember. I think we went through peaks and troughs of being better or not. As long as we had somebody who was actually always there and their focus was on reminding you, which was quite useful when you had the research nurse, because they absolutely remembered. It tended to happen if you had a trainee who was on the ball as they went through the ward round bits on the CCIS, but there were occasions where it slipped through, and there were occasions where I even recall actually reminding some of my colleagues, although I’m not suggesting that I wasn’t guilty at times of forgetting and then of having to be reminded.”* (PICU Consultant, S04)  *“I would say it’s being implemented well. Definitely the consultants that are on here say have you done an SBT today, you know, are we going to try an SBT overnight, they’re quite hot on it. I would say, a step down from that, the registrars, they’re supposed to set the comfort parameters that we’re aiming for with each patient, which they don’t often do, so you don’t know what you’re aiming for…Too much to do. They’re on a ward round frantically typing in…”* (PICU Bedside Nurse, S04)  *“It’s tricky to remember. We had the cohort of the first trainees [registrars] that were here six months just when Sandwich started. They were very good at prompting us during the round to ask and fill the gap, but obviously as you get more recycling it fades, and again, you can tell people just please remind us, because we are making plans of ten different things. That doesn’t happen much.”* (PI, S04)  *“I don’t think it was routinely discussed with every patient. This is something that kind of comes into conversation if it’s an issue [with a particular patient]. I don’t think they’d come around on ward round and say “oh, we’re targeting 12 to 17.”* (PICU Bedside Nurse, S06)  *“I think some consultants and some of the doctors did that, and then obviously some got kind of missed. It depended which consultant, that’s what I found…”* (PICU Bedside Nurse, S14)  *“I think when the research nurses were on the ward round and they reminded us, we remembered to do it, but when they weren’t then we don’t always remember.”* (N/PICU Consultant, S08)  *“I think it’s got really embedded because of ward rounds, because this is the chart that they're supposed to fill in on ward round to say did we do SANDWICH…”* (PICU Senior Nurse, S15)  **Sub-theme: Development of an underpinning momentum towards extubation**  *“… especially from just starting a year ago when this was all coming in and stuff, for me, yes, definitely to look out for signs when a patient can be extubated and, yes, as you said, to look for signs of being over sedated, when a patient doesn’t need it to actually…”* (PICU Bedside Nurse, S02)  *“So but I thought it was a good…the comfort scores and the ventilation and trying to get them weaned and trying to get them off and just kept…I think there’s been a bit of a culture where" well we’ll review it tomorrow," whereas your main aim on the night shift or on the long day shift was to try and get them to this SBT and then there was thoughts then to prepare for is this patient a candidate for extubation … It definitely made us think ahead.”* (PICU Registrar, S02)  *“I think from filling in the SBT chart it gets into your head, like you come on shift and you look at your patient and you think, actually, like they would suitable for an SBT. So like it triggers you to ask the doctors.* (PICU Bedside Nurse, S18)  *“But what we found was if they failed the SBT, instead of going back to the pressures they were on, we went back halfway up, so even that helped. Okay, maybe they’re not ready to go down from 20 to 5, but okay, they want that back, take it up to 16.”* (PI, S18)  *“It was definitely sort of a catalyst to get people to think about weaning. The kind of experience that we get on wards when we go and see patients and you look at their charts over 24 hours and they’ve been in the same amount of oxygen for 15 hours, and their sats have been 97 and above for that whole time, it doesn’t look like anyone tried to actually wean them, and you get into that rut where it’s easy to leave things as they are… So just to know that there’s actually a process in place to actually think should we be looking at extubation or weaning to extubation or not, I think has been good for a lot of people … “So I don’t think it’s radically changed things, but it’s certainly allowed greater efficiency in terms of trying to get these patients off the ventilator as quickly as possible. … it feels like that process works for considering every time, every opportunity to consider when to do these things, even if you say no.”* (PICU Physio, S18)  *“Overall, I think it's been really, really good. It's changed the mindsets, it’s changed the language, it’s allowed people to positively challenge the way we work in a "let’s try something new approach."”*  *“Yes, because it made people question and think, as opposed to just going along.”* (PICU Senior Nurses, S03)  *“I think that’s the benefit of most of these trials actually. Personally, and speaking honestly, I think the direct impact of any SBT, or whatever the design trial, is quite hard to pin down to individual patients, in reality, but the knock-on effect of just focusing everyone’s mind on one or two issues, I think is really beneficial.”* (PICU Consultant, S03)  *“I think it pushes you to wean, it pushes you to at least think about weaning. So if you don’t go on to five and five, at least you’re doing something.”* (PICU Bedside Nurse, S04)  *“Well, we say it’s four hours before extubation, if you know they’re going to pass, if you know they’re going onto an SBT then you start fasting at that point.”* (PICU Senior Nurse, S04)  *“What I found useful is that it makes you think about extubating, it makes you more proactive, rather than just waiting for the baby to do something to decide on extubation. So that was the main thing for me, being proactive about thinking that this child may be ready for extubation.”* (PICU Registrar, S13)  *“The patients who it benefitted, it really helped, because it helped them move along quicker, and because we were trying harder, it moved them along quicker...the patients that it was designed to help, it did, and I think that was good and if nothing else I think formalising the discussion on using a score and saying are the patients compliant, are they in a weanable position, I think that was quite helpful.”* (PICU Senior Nurse, S01)  *“But when the doctor’s done their ward round, they'd normally come around at the start of a nurse [night] shift and say probably keep this patient nil-by-mouth from 4 o’clock [am]….this patient will probably pass [the SBT] because of the type of patient that it is.”* (PICU Bedside Nurse, S01)  *“Yes, sometimes it [SBT screening criteria] wouldn’t have been what I would have chosen as a readiness for extubation, as in the limits were quite high. But it did make us think about readiness for extubation earlier, I think, you know, having those wide, and then if they went on to have an SBT and passed it, it probably pushed us more towards thinking that someone was ready to extubate, when previously we'd have said they’re not ready. If they were on 22 on 6 or something we wouldn’t have pushed them maybe.”* (PICU Consultant, S16)  *“For me as a manager, it gave me an overall surveillance in monitoring what was happening at the bed space. You could plan the patient's discharge, to be honest with you, and it could expedite a lot of things. For example, when you get them off the ventilator, the sedation, wean, what steps to put in place, how to manage them and where to step them down to. I think that was the whole…all of the information was always on it, it was a daily narrative and it became so engrained in what the medical staff and the nursing staff did, and that was always fed back into our planning meetings about when we can plan for discharge and when we can plan for extubation, and basically as a manager, I wanted to know when my next bed was made available, and it came to me every day in one simple format and one sort of one line of communication, if that makes sense to you…”* (PICU Ward Manager, S16)  *“And also being a bit more prepared as well.* *If you know that your patient has passed the SBT and we're most likely going to extubate, then I might think okay, let me go and get a non-invasive ventilator or some oxygen or whatever so you're prepared…”* (PICU Bedside Nurse, S15)  ***“****So again, I think if you had a senior nurse who's confident, and the doctor was happy, you might stop the feed, because that's quite a thing that sometimes doesn’t happen.”* (PICU Senior Nurse, S15)  *“… we knew they’d passed their SBT, we could then get on with extubating, rather than discussions at ward round, actually, looking at those pressures, this child’s ready for extubation, but actually we had stopped the sedation, we had stopped the feeds… So they were being extubated mid-late morning rather than early afternoon.”* (PICU Research Nurse, S07)  *“But I think that helped them to see where their patient was in terms of you may think that they appear like they may be ready, actually, no … they’re definitely not ready, they need…but maybe they don’t need as high a support as they were on. So some of them were failing but not going straight back to the same settings. So it had still encouraged them to wean, and I feel like if the prompt for SBT wasn’t there, maybe that wean would have been slower, because prior the weaning was they're on 10, let’s drop them to 8, so it was very slow. Some of them definitely did require a slow wean, but for some patients you could have… definitely gone to CPAP a lot quicker. I think you did see the change in that.”* (PICU Research Nurse, S03) |
| **Theme 4: Promotes consistent weaning related care**  **Sub-theme: Enhanced prioritisation and conduct of patient sedation**  *“What I do think influenced people’s actions on sedation was their assessing readiness for SBT. So if they would frequently come to a patient that was ready, all except for not awake enough, that was what was eye opening for people. Why have we got to the point where this patient is ready for SBT, aside from the fact that they’re not awake, why have we got to this point.”*  *“It puts sedation on the map, which is something that I’ve battled on and off with for a number of years. So I know that even if Sandwich doesn’t work, again, it’s going to raise the profile, the correct sedation and getting comfort scores right … “… I definitely think it’s really been very positive. And also thinking about other types of sedation, you know, enteral sedation. Do we need to give them IV sedation? I think it’s kind of opened up a different way of thinking.”* (PICU Senior Nurses, S10)    “*I think really important, because we’ve always used comfort but it’s been very much a tick box exercise and I don’t think anyone really thought about what it meant, how to use it, it was just another thing you had to do, and I think this definitely made it a little bit more tangible in people’s minds, "oh actually we can use this as an assessment tool, not just a tick box... So we did our own internal audits on sedation use, pre and post-intervention, kind of very crude and just looking at how many extra boluses we were giving, what was the maximum comfort score of these patients that have received lots of extra, that type of thing, and those results were massively improved, so it was by like 67% our use of sedation was improved.”* (PICU Research Nurse, S10)  *“I think before we were doing the comfort score, some people were not doing it properly… [but now] for the ward round and then it's prompted, should we wean and all this stuff, and if you have kind of some senior person there then they will be more kind of proactive to do sedation and all that stuff.”* (PICU Senior Nurse, S16)  *“… before Sandwich, although we’d use sedation scores and comfort scores, we hadn’t done it in a systematic way… so it made it much more as a routine part of the ward round which is important for us, because our sedation practices were not the best, I don’t think… in terms of our assessment of sedation and what we did about it. We never really did it in a systematic way. So this was good. It introduced a systematic approach to sedation for us.”* (PICU Consultant, S16)  *“I’ve been here ten years, it taught me a lot about comfort, and actually, I probably, in my clinical days sat with patients on 18/20, it seems fine. The understanding that Sandwich brought in, they’re hypersensitive, they're too awake and you potentially need more sedation because of your boluses and you’re not going to be able to wean them effectively. I thought that was really good...”* (PICU Bedside Nurse, S01)  *“I think Sandwich brought a lot. Sedation and ventilation weaning in children, for me, it taught us a lot about sedation. It wasn’t just about how much sedation a patient be on, it brought in nicely about pain and you should always be on a pain score.”*  *“We were originally doing Comfort B, we were already Comfort B anyway, but we were doing it our way….You’d look at a chart, you can see, it might change at 7 o’clock during handover, pretty much guaranteed for a lot of patients that number stayed the same all day.”* (PICU Research Nurses, S01)  *“…it kind of had a bit more purpose once SANDWICH was…in motion, because I was just like "why are we doing comfort score, what does that mean?"* (PICU Bedside Nurse, S15)  *“I think it prompted me slightly more. Personally, I did find it did prompt me, like especially if I had a very busy patient… I’d think, oh actually, I haven’t done a Comfort B. So it was making me think a bit more…”*  *“… what I did like about the Sandwich… is looking at the pain aspect. So not always going for sedation. So when you’re looking at like your Comfort B... you know, well, are they actually in pain, instead of do they need more boluses or sedation or would actually just a bit of Paracetamol help just take the edge off? I think that was a really good thing in it.”* (PICU Bedside Nurses, S07)  *“I think using pain scoring in combination with comfort scoring has sort of been highlighted by a lot of the nurses…as a good tool. Yes, because I can see whether I need to put the sedation up, morphine etc., because of pain or whether actually they’re adequately sedated, and actually a lot of our children who are with us for a while go onto withdrawal, which is a massive problem, and if you get the weaning process better then we’re avoiding that, and that’s always a good thing.”*  *“I think the comfort scoring itself was fantastic, and we’d do pain scoring at the same time, so we could then compare it to see whether the patient was adequately sedated or whether they needed any analgesics.”* (PICU Research Nurses, S14)  *“I do think it's made people more mindful…I have noticed that perhaps sort of we’ve been more conscious of not over sedating patients and perhaps trying other avenues to prevent the patient becoming over sedated.”*  *“If I saw a really high comfort score I’ll be like what’s wrong. They obviously need something else, so it would make me think. I’d definitely look oh that baby’s really flat, like I’d maybe want to come down on something. Yes, it does make you think.”* (PICU Research Nurses, S14)  *“I liked it from the nursing point of view of the comfort scoring, pain score meaning something and being useful.”*  *“I mean again, it is subjective but it made us think about the sedation and all that stuff and how it’s affecting the patient.”*  *“…I really liked the comfort score and the sedation score and pain score, you were thinking properly about what your patient needed and whether you were over sedating or whatever. I feel…definitely, our patients are a lot more awake [and] on sedation for less time. I don’t know if that’s due to other changes in the hospital but I feel like I’ve definitely seen a difference from when I started.”* (PICU Bedside Nurses, S03)  *“Because we are very obsessed with having a very still patient in the bed, so actually with the comfort score and being able to wean that sedation just helps with the SBT and just getting them off the vent… I think it [Comfort Scoring] definitely triggers people to look at it and think right, well, I could start weaning…or not even nurses, because nurses still like a very well sedated patient, but it will trigger the doctors and the ANPs to think “oh I could reduce that morphine”. Or, even us as 7s, when we go around, to go "do you think we could wean on that?", and we’ll have a discussion and then we can wean on sedation.”* (PICU Senior Nurse, S18)  *“Yes, well we did comfort score before, but this has definitely ensured that people do it more regularly, and we listen to comfort scores and act on comfort scores a lot more than we did previously…. in ward rounds previously, consultants may not have said what is your comfort score, they’d have just said are they sedated, are they not, but [with SANDWICH] they’ve been able to…say …are you 12 to 17, so then they’ve got more of an awareness and then it’s given us something to focus on a little bit more than kind of saying yes, they’re sedated enough or no, they’re not sedated enough.”* (PICU Research Nurse, S18)  *“[Before SANDWICH]…they wouldn’t be coming at us asking for comfort scores, they’ll be happily having somebody sedated up to the hind tooth because it made life [easier]… my reason that I wanted a Sandwich trial wasn’t necessarily because I believed the Sandwich trial was going to make a difference to the world, but I did want the unit to have comfort scoring embedded in their everyday practice, and despite my best efforts and the best efforts of many, I haven't been able to make it become a routine habit that the nurses would be noting down comfort scores and acting upon it. So I wanted something where you get that good practice, and that’s happened.”* (PICU Consultant, S18)  *“And they’re better at pain scoring as well. It goes hand-in-hand, doesn’t it? Because in the training pack of the Sandwich trial it says that if this score is higher then it’s more of a sedation issue, if this score is higher then it’s more of a pain issue. So that, in a way, would allow you to determine which to do first or how to intervene, basically.”*  *“So our targets were set by our consultants in the morning, so we always knew what we were working towards. I found it really useful, because I have no previous experience before the Sandwich trial, so with the Sandwich trial I was able to score them and wean their drugs, which before I wouldn’t have been able to do with my own initiative.”* (PICU Bedside Nurses, S06)  *“I quite liked having a number, because sometimes you think that they’re maybe too… they're well sedated, but they may be over sedated. You could see that by the numbers, which was good.”*  *“…encouraged us to do the comfort score to know what is our target, because without that target we don’t know if the child’s been wiggling all day, you know, but with that you can give them a number to focus on.”* (PICU Bedside Nurses, S02)  *“… overall, it was really good, because it meant the nurses were looking at sedation properly, we were looking at it properly and it was being discussed on each ward round.”* (PI, S16)  *“I think it was good. It made you think more about sedation and whether they were over or under. You could categorise it a bit better and say we want this target for this reason. You had more clarity.”*  *“It definitely made you talk about the sedation as a whole, didn’t it? How sedated do we want this patient?”*  *“I thought that was important from a sedation point of view, because we weren’t probably the best in thinking about weaning sedation...it was four hourly that we were thinking about, whether we could wean sedation, and we weren’t doing that before, it was mainly 12 hourly, 24 hourly, which is slow. So yes, it definitely prompted us to think about sedation a lot more. Before it we’d had patients that were probably ready to extubate but then were on too much sedation to be able to do that.”* (PICU Bedside Nurses, S13)  *“I think it’s good in a way that it makes you think about it at least twice or thrice in a day, morning rounds, evening rounds and then for the person looking overnight. So at three points in time you do seriously think about am I over sedating? Do I need to titrate a bit? Do I need to come down? So it is helpful, I would say.”* (PICU Registrar, S13)  *“I felt like it focused the nurses' minds on weaning sedation more quickly, so I used to forever get frustrated that I would be forever saying "please can you wean?", and I'd be weaning the ventilation but they'd just be took sleepy and I'd come back and say carry on weaning, or I’d say can we stop it, whatever, morphine or medaz, and the nurses might have been a bit reluctant, for whatever reason, to do that. So I felt with this, at least there was a weaning protocol and that they should be doing their scores and then weaning on whatever the comfort scores were.”* (PICU Physio, S04)  *“I think it was good for getting a culture of getting the comfort score completed more regularly, but I think there’s still a theory practice gap between performing a comfort score and acting on a comfort score. So like I said, I think we have been doing it and actually we have got used to children being more awake.”* (PICU Clinical Educator, S04)  *“… the sedation is good, is really good, it makes everybody think carefully instead of saying just continue with the morphine… So I like that, and I don’t know for a fact, but my instinct is that if the child is comfortable and not zonked out then they are going to extubate earlier. So that to me is the primary thing. All the others, they’re processes, but the fundamental thing is the child is not too zonked out … I do like the idea of saying “okay, this I want to try at 17 or something.” That’s a clear number, I know what the number means, the nurse at the bedside knows what the number means and we can work towards it.”* (PI, S17)  *“…I think it was a lot better. We did tend to use oral sedation a lot more insomuch as we would ring them in, in order to take them off and we wouldn’t just let them withdraw, and I think that’s still the case now, that that has carried on from that…I think that side of it has, definitely, from a comfort point of view, that we don’t seem to have as many children that would necessarily be withdrawing. We do something about it.”* (PICU Research Nurse, S17)  *“…and there was a bit more encouragement of giving some enteral sedation so we could get the high sedation down, which I think definitely started more after Sandwich.”* (PICU Bedside Nurse, S13)  **Sub-theme: Impulse to adhere to preferred sedation practice *(also addressed in ‘Enhanced prioritisation and conduct of patient sedation’, above))***  *“I still think they’re over sedated, because, there’s a terrible fright that they accidentally extubate, which, without any doubt, is dangerous, and there is literally a phobia in using other ways of making sure that patients don’t pull their tube out, like muffling …”* (PICU Consultant, S16)  *“Yes, so I think maybe the prompts, the questions they ask to give a comfort score would then be like okay actually, maybe they are [oversedated]. But I don’t think everybody would act on it without being told by the nurse in charge…It's just if they’re more junior.”* (PICU Senior Nurse, S16)  *“I would have said that pre-Sandwich, people did a comfort score when they felt their child was wild and awake because they wanted to have the evidence to rationalise increasing sedation, but if the child was flatter and asleep then people didn’t score, because the child was comfortable and asleep. I would say when we first started Sandwich everybody felt a bit uneasy, because to lots of people it felt like we were keeping children more awake than we were used to keeping them, and particularly when the unit was busy, that unnerved lots of people because it makes the acuity harder to manage… People are very good at scoring but I don’t think it gets acted on… So I think there was still an element of reluctance.”* (PICU Clinical Educator, S04)  *“… but when there’s sick patients, the really, busy patients, all the girls would just say “I’m too busy”. So that’s as a last thought, instead of thinking it should be included in everything…”* (PICU Research Nurse, S07)    *“For me, it wasn’t an issue. However, there may have been times on very busy days that I… maybe didn’t do it as frequent as I ought to, but as the time went by I think I made a bigger effort to try and fulfil the requirements…”* (PICU Bedside Nurse, S01)  *“But they wouldn’t be coming at us asking for comfort scores, they’ll be happily having somebody sedated up to the hind tooth because it made life [easier]…”* (PICU Consultant, S18)  *“I did find though, some of my patients is there…I mean granted, a lot of the ones that are sicker, they’re not eligible for it anyway, but there were the odd occasion where I was just too busy to even look at it sometimes.”* (PICU Bedside Nurse, S03)  *“Since I’ve worked here it’s always been a sedation heavy unit. So completely changing our kind of weaning practices was one thing, and people were a little more on board with that, but changing sedation practices was always going to be a struggle and we’d gone through numerous sedation guidelines and people [prefer patients] … Sleepier, yes. 100%. And that’s coming from our senior nurses, because they’ve always done it this way, they’re used to quite flat kids… we’re a trauma centre and we’re a neuro centre and a liver centre, most of our kids are ventilated to neuro protect or to facilitate like dialysis, those kinds of things…so because of our patient populations we tend to be a little heavy handed with sedation, so when it comes from our bronchs that could be a little bit more awake…I think it made people think more about it… now thinking "maybe it's pain, let’s try and fix that first, let’s give a smaller dose of morphine to see if it’s pain, oh they’ve settled." It wasn’t that they were awake, it was that they were in pain and those types of things. So I think in that regard it’s definitely made a difference with how much we give extra but in regards to all their comfort score’s been consistently 12, let’s wean something, that’s [more difficult] …”* (PICU Research Nurse, S10)  *“The main thing that stops it all having the impact that it can have is the culture shift, and a trial can’t entirely manage a culture shift. There are still a lot of nurses that want more sedation and that’s bigger than any trial.”*  *“I think there’s a massive shift in culture. I genuinely think there is. I think it’s really raised the profile of it [sedation]…”* (PICU Senior Nurses, S10)  *“Sedation. I like the sedation idea because it does two things. One is it gets us all agreeing on what the sedation target is. The other things is over many years you get to know your nurses quite well, and some nurses like their patients well asleep because they can sit and just have a quiet night, and some don’t. Here we can try and standardise that…if we can get the nurses more confident about using a combination of different oral sedation, in addition to the main IV ones, then I’m happy for them to juggle it, because in the end, they’re the guys who have to hold the tubing when the kids try to pull it out….Like all changes in medicine, it will take time and go backwards and forwards, it will never be a nice smooth thing…”* (PI, S17)  *“I do personally like a well sedated child. People say because it's easier, not just because of that. I think as a bedside nurse, it can be really hard when you’ve got an awake child and sometimes that line is very fine of getting sedated enough that they’re awake to a point of being able to extubate but being settled and sedated, and so you can’t turn a child sometimes, they’ll be fine and they look fine but you turn them and oh, my god, they’re awake.”* (PICU Bedside Nurse, S17)  *“I think it’s given the Band 5 a bit more structure. I think they were a bit scared about sedation. They would like the patients to be completely flat…I think it’s given them the confidence to have a slightly more awake condition…”* (PICU Senior Nurse, S13)  *“A mixture of things. I think (1) because it wasn’t followed and (2)… we should be able to adjust the sedation if we needed to. If they passed the SBT we should extubate, not because they’re not opening their eyes or not awake enough… that’s a consultant decision…Because of that…we ended up waiting all day, until 4 or 5 o’clock in the evening, and I had to keep pushing like this child…sometimes you have kids where they just don’t wake up or open their eyes and you just have to take that chance. We have done that before with a previous patient. So yes, that didn’t make it happen any quicker.”* (PICU Bedside Nurse, S10)    **Sub-theme: Expediting patient extubation**  *“I enjoyed doing it, I think it was really good to have a plan, and also it worked, that you didn’t have to be on a 12 on 5 to be extubated and there was certain things that were drilled into us that we thought they had to be on these very low pressures for you to successfully extubate, and that’s not the case.”* (PICU Bedside Nurse, S10)  *“…when we got the bundle I was like we’re going to have to use so much more non-invasive because the pressures are just so high, we would never extubate or even think about extubating anyone, like we’re talking maybe 24 hours later with pressures that are that high. But then some patients passed and extubated and were fine. So I think it took people to see it happen in real life to think “oh, okay”…* (PICU Research Nurse, S10)  *“The SBT screens are great, because it focuses your mind and makes you really question whether your patient’s ready or not. The weaning from a PEEP of 22 to a PEEP of 10 on CPAP, that is massively different to what we would have done before… Right the way down to 14 probably, or even 12, and then gone onto CPAP.I think that’s really important. I think the SBT screen has been really important to focus people…”* (PICU Research Nurse, S18)  *“I mean, I think the good thing about it, it pushes people to think about sedation and what we’re doing with sedation and how we’re weaning them, and actually what you find is you come in in the morning and they’re actually on a Spontaneous Breathing Trial already…”* (PICU Consultant, S18)  *“I think the concept of trying to improve or expedite extubation was good. I think that we were probably very set in our ways before and this questioned and challenged us to look at different ways of working… and focused on weaning and can we do it quicker and can we do it more efficiently, and I think it had a big impact on people, considering that and actually considering a child could they wean earlier and probably expedited weaning in a lot of children.”* (PICU Physio, S03)  *“I would say one of the biggest things I noticed was that we were weaning the ventilation a lot quicker than what we would have done originally. So having the SBT there as a prompt, you were then on to 5 on 5 way quicker. It almost felt sometimes like ooh that’s a bit fast, but actually the patient was fine and probably then did get extubated quicker.”*  *“I think it prompted you a lot quicker to do a lot of things.”*  *“In terms of priority, it did go up. I couldn’t say if it happened any quicker.”* (PICU Bedside Nurses, S03)  *“So I think it changed our mind, saying "okay, so it can be extubated, there is no reason why we shouldn’t try," and then of course you assess clinically but at least you have the mindset of saying "maybe, why not?"* (PICU Senior Nurse, S15)  *“I think, on the whole… it probably did help, because it pushed us into doing SBTs, us and the junior nurses into pushing kids onto SBTs quicker…then if you’re having people put them onto SBT quicker and naturally maybe they are able to come off [the ventilator]… having been quite far away... It pushed us to wean quicker.”* (PICU Consultant, S01)  *“But one of the things I would say is I think I’m probably not alone in being surprised by how soon we could extubate some babies, because you progress from 20 on 6 or whatever it was, 20 on 8, right down to a Spontaneous Breathing Trial, and then you think oh, I’m not sure they were ready, and oh, where they’re ready, you have to stop the sedation… Yes, so it caught you out unawares…”* (PI, S18)  *“Yes, because it made people question and think, as opposed to just going along.”*  *“I think ultimately it has sped up the weaning process.”* (PICU Senior Research Nurses, S03)  *“Well, we certainly had a cardiac patient… [who] progressed incredibly quickly because Sandwich was being followed, and I think I came on at half four and he had passed an SBT and been extubated and I remember thinking wow, and not being sure whether it was a good or a bad thing, because the magnitude of the cardiac surgery was such that this was never a child I would necessarily have anticipated being extubated within that short period of time. But that said, the child was okay. So I had to challenge my own pre-conceptions with it.”*  *“I certainly saw a few times taking a child from what we would consider full ventilation to an SBT without any weaning process in-between, which is often what you want to do in practice actually, but it just feels a bit rushed to do that sometimes, and before Sandwich you can often feel that staff at the bedside were thinking "well, what are you doing?" And the fact that we were able to do that in this trial, occasionally successfully, was helpful. I thought that was the best.”* (PICU Consultants, S03)  *“…yes, and I do think the SBT is quite good actually. It’s not something I’ve never used before and I feel like probably more patients we’ve extubated…*  *Off the back of that. [speaking together]*  *I feel like that.”* (PICU Bedside Nurses, S17)  *“… and I enjoyed the SBT, the ventilation trials. I think that was a brilliant idea… and certainly it helped me in deciding which patients I could extubate earlier. Put it like this, so if it was done by either early morning shift or by the night shift then I knew okay, this patient has passed it, and the only time I would not have done it, if it was something else i.e. patient needed a blood transfusion.”*  *“… I used to not drop it to the levels we’re using because I thought it would be too dangerous for the patient, I will be honest with you, and now I'm convinced it actually isn’t that low, so I'm happy to have it on those lower settings and I felt it could have possibly been my previous conceptions were wrong.”* (PICU Consultants, S17)  *“I don’t think it influences the 9 o’clock decision very much, but what it does do is it allows us to think, hang on a minute, we’re not ready for 9 o’clock, let’s try the 1 o’clock one, instead of saying well let’s see what happens and then you leave it and then it comes 5 o’clock and you think oh, 9 o’clock/10 o’clock tomorrow. So I think definitely the ones that you may not have pushed to get extubated between 4 o’clock or so, the 1 o’clock assessment thing is a difference.”* (PI, S17)  *“Yes. I think they were quite up for that, to make sure that they were ready, to do this 5 on 5, which obviously when we first had our meeting for the training, some of the consultants were a bit iffy about it and I think, as time went on, they probably got to see the point of it, and I think the nurses definitely did, to be honest, I think we could see it, and I think sometimes the nurses feel a little bit more no, my patient’s not ready, I don’t want that to happen… I think that’s [systematically weaning down on the ventilator] what’s always happened, yeah.”* (PICU Research Nurse, S17)  *“… because it enforced you to think about certain things that wouldn't be routine, should be routine but weren't the first thing you'd think of, such as where would you want your sedation level to be, for example, or saturation targets… Has it inherently changed the culture, probably, because that's been a routinely part of my checklist that wasn't before. So I think it has made a difference, from that point of view. The additional aspects of being challenged as to if SBT hasn't been passed, for example, why aren’t we extubating or why aren’t we trying an SBT in these patients, again it allows, which I think is quite powerful, empowerment of the bedside nursing staff to say "why aren’t we doing this?"* (PICU Consultant, S04)  *“I’d say we’re much more proactive now at putting children on spontaneous breathing tests, rather than the age-old process that you used to just wean rates by five and check pressures by silly little amounts. So I’d say we’re much more proactive now. If a child’s ready to wake up, just cracking on and reducing sedation and getting them extubated swiftly.”* (PICU Senior Nurse, S04)  *“I guess the bit that worked well and why I agreed to be the local PI for the studies, it has increased awareness that children can go straight onto spontaneous breathing trial, then get extubated much earlier than they would what general people think…”* (PI, S04)  *“I think it sped things up, I think we sort of pre-empted extubation a lot quicker, I felt.”*  *“Yes, it made you just think about it, like you would usually think “I don’t know if they’re ready”, but actually if they ticked all those criteria then it made you consider patients that you might not have considered.”* (PICU Bedside Nurses, S13)  *“I think the answer to that was I was surprised how often it was okay. So they [“5 on 5” settings] were lower than I would have expected them to be, but just using them in the trial has made me think most of the time that’s okay. One of the interesting things was that I wouldn’t really have expected to extubate people from 45%, that was possibly more than I expected, so I would usually have it below 40 before I thought of doing it … But also, to some extent, that was almost the point of it, was that it meant that you didn’t need to do all that weaning, because what we’ve done, we got to the parameters where you were within the thing to jump, and that was partly what changed my mind and that’s why I think certain patients that I had would maybe have taken a day to wean them, I actually just went, "well, they’re in the parameters," and just go to the SBT.”* (PICU Consultant, S02)  *“For example, I think the SBT is a brilliant concept, an absolutely brilliant concept, and I actually did it on a patient with someone who they were just adamant wasn’t going to extubate, and I was like let’s do it, let’s do it. Then they extubated and I was like see. I was like trying to prove to people that I think the SBTs is (a) really safe because you’re not having to like whip a tube out and then whip one back in. So I think the concept is brilliant. So I really wanted, if anyone took anything from the unit, it was that. I was desperate for people for that.”* (PICU Research Nurse, S14)  **Sub-theme: Impulse to adhere to preferred ventilator weaning practice**  *“… that’s probably the biggest thing actually, isn’t it, the having to go to 10 or 5 on CPAP, and that’s probably put people off, as in consultants. So sometimes when you pass your SBT screen but then it’s still a conversation as to whether you do the SBT.”* (PICU Research Nurse, S18)  *“A lot of people wouldn’t do the 10 on 5 either. They wanted higher CPAP pressures. I didn’t really understand why we wouldn’t at least try the 10 on 5 and then if we fail then we could maybe try 12 on 6 or whatever. There was no even attempt at that with some of them.”* (PICU Senior Nurse, S18)  *“I think the biggest problem we have here with mode of ventilation is that people leave the apnoea alarms very low on spontaneous mode, particularly the babies, they would get 15 seconds before they’re having an apnoea, and then they’re failing, when actually, if you put it to a realistic amount, you can allow them to pass. So I think interpretation of those parameters and the interpretation of what the machines will do and what it means to have an apnoea or an alarm and things like that aren’t necessarily there, so that underpinning knowledge of some people.”* (PICU Physio, S18)  *“But I think sometimes we’ll flip them to SBT and then, within the first minute or two, they might apnoea because they’re just not used to it, and we’ll think oh, another fail, switch them back. I think some doctors are really quick to switch them back, whereas others will ride it out a bit longer to see if they’ll manage, and it’s whether there’s a minimum time that they need to be SBT, unless it clinically deteriorates. Sometimes I feel like the doctor will stand over them and watch and watch and watch and if something on the numbers, the apnoea, the other alarms are going off, they will just turn them back straightaway without giving them the chance to actually get into the rhythm and breathing for themselves… I think when my patient did like the apnoeas we gave them three times until we flipped them back…because they would do it and then they would sort themselves out… It’s different doctors do different things.”* (PICU Bedside Nurse, S18)  *“… well some of the consultants come round first thing in the morning, but they'd just change the settings, they wouldn’t necessarily have looked at that chart [for SBT screen result]… So they went down and put their rate down or put them on to CPAP anyway, regardless of what the score was…so you would have come behind and done the score after so that you could fill in… They wouldn’t necessarily have actually looked down.”* (PICU Bedside Nurse, S02)  *“You’re looking at a Peep of 8, for some of our kids 8 still quite a significant Peep. Like if I saw Peep of 8 I would think this patient’s actually quite sick. I wouldn’t be going thinking we’re going to do an SBT on 8. And the same with the peak pressure. So 22 and less, I just felt sometimes it was a big jump, because actually in my mind, I’ll only really truly start thinking whenever they are less than 20, you know, sort of 18/16 that’s when I am gearing up for a CPAP, a trial …* “*We did implement a lot of modified SBTs… Seven on Seven or, you know, and that was considered an SBT, do you know what I mean, where it wasn’t your exact criteria”.* (PICU Registrar, S02)  *“There were patients, who for complicated reasons you said “right we’ll just leave them on the settings until tomorrow. We’re not going to extubate so don’t change the settings.” So the two hour window, I think it’s probably a good thing to have, in that you don’t want people not having decisions about things, but I hope in most ICU’s it’s sort of irrelevant, I hope they make decisions before the two hours… What we often did was if I said right, the SBT's passed but I don’t want to extubate for a couple more hours, I’ll put them back on their other settings to make sure that they don’t tire and they we'll get extubate at 6 o’clock.”* (PICU Consultant, S02)  *“I think the bit that didn’t work was the Spontaneous Breathing Trial, because people either forgot or the doctors would come round and just extubate them just casually”* (PICU Bedside Nurse, S14)  *“There were certain patients that we felt that the SBT wouldn’t work. Cardiac patients were very fragile, so they'd meet the criteria, so sometimes people would say “just don’t do it because we really don’t think it’s safe”, and by the time we'd do it they wouldn’t tolerate it well…We would say something like "okay, so if this patient meets the criteria later with the SBT, can you not do the SBT." So we try and have a discussion and make an active decision not to do an SBT.”* (PICU Consultant, S11)  *“Some of the time it was a clinical reason or there was some other plan in place. Some of the time I just personally felt it was a clinician thing.”* (PICU Bedside Nurse, S03)  *“If they were on a rate and then we were going to put them on 5 and 5, they were actually really good at dropping it quickly, that wasn’t the issue, it was the number [continuing to use “6 on 6”] that was the issue.”*  *“Yes, because for so long everyone’s been doing 6 and 6. They were doing the Sandwich checklist but still doing 6 and 6, not 5 and 5.”*  *“Yes. So that was the hardest thing, getting people to change to 5 and 5.”*  *“And to explain why… it doesn’t count as the same.”* (PICU Research Nurses, S03)  *For me, it was just conceptual, so I didn’t really mind if it was five or six. If someone left it on six I think I even allowed it a few times and just said well, effectively we did it.”*  *Yes, I couldn’t see a lot of difference between the six and six and five and five. I do find that I’ve crept back to six and six. That’s just years of having done it but it doesn't mean I think it's any better or that I had a problem with five and five, it’s just language that you’re used to.”* (PICU Consultants, S03)  *“… I’ve noticed some people [bedside nurses] will turn it off straight away, so if I’m in charge and I’m on ward round, they’d be like “oh they failed” and you’re like “how long?” and they were like “two minutes”. You’re like can you really fail in two minutes? I don’t know… So they’d do the screen and they’d tell the doctor and then they’d come and put it on, but then if they [bedside nurse] felt they had failed sometimes they would tell the doctor and the doctor would say “oh, go back then”, but I don’t know whether they’d do a full assessment, especially after a minute.”* (PICU Senior Nurse, S16)  *“There’s still a lot of reticence from, considering how high the pressures, how high it had been set, at the point where you could do the spontaneous breathing test, I think our overall position, as a unit and the nursing staff, was they felt more comfortable waiting until the patient was on less than that before going for a spontaneous extubation.”* (PICU Consultant, S04)  *“But then sometimes I’ve had it, if they passed the screening they don’t fully put them on an SBT, they put them on a half SBT and they do like a slow wean from there. Rather than doing a big jump and they know they probably would fail it, they do like a slow wean on that, if that makes sense. So if they passed it with a PEEP of eight, instead of going straight down then to five they wean them down to, let’s say, six and go then to five … It’s like sometimes they’re not really put on the five and five. Do you know what I mean? So then I was like do they technically pass…so quite a lot of the time it’s not really a true SBT. They do finally end up going on to it. When they’re meant to go straight on to an SBT, they don’t.”* (PICU Bedside Nurse, S04)  *“Well, if I’m honest with you, I would do the SBT, check the gas, look at how they looked and then, whilst I was getting everything ready, I would just hold them on the bag, and it must just be a comfort thing for me, I don’t know, because you just need to be sure.”* (PICU Physio, S04)  *“I don't think any of these [patients] jumped from 20 to five…It’s too much of a jump. … That’s not how we work at all. So it’s been yes, let’s take the ventilation down…“It’s used as an extubation predictor. So when we think the child is ready to extubate, we do the SBT … So there’s a complete bias in the way we use the SBT.”* (N/PICU Consultant, S08)  *“Again, patient to patient, some of the patients aren’t capable of…if they were extubated and on non-invasive, they wouldn’t be on 5 on 5. So then you couldn’t do an adequate…like you couldn’t do the protocol version of the SBT because you’re almost setting them up to fail because you’re giving them less than what they would have normally.”* (N/PICU Bedside Nurse, S08)  *“Sometimes they just ticked a box and you don’t quite know, but I felt like probably that majority of them were genuine.”* (PICU Research Nurse, S13)  *“Maybe the hardest thing was, because we weren't putting people onto CPAP like that routinely, we would do, but we'd already made the decision that we were going to extubate and then we'd put them onto CPAP for half an hour or something just to prove that they could do it before we extubated. Doing it the other way round is obviously…”* (PICU Bedside Nurse, S13)  *“When [name of research nurse] and I would go collecting data, we’d see 6 on 6, and you’re like “why is it 6 on 6?” and they’re like “just because it’s really low, 5 on 5, for them.” It was just random and you’re just like “well I can’t say that’s an SBT now because it’s 6 on 6.””* (PICU Research Nurse, S07)  *“Today they said do the trial 5 over 12, so it depends…If they’re planning to extubate to non-invasive…we’re not going to extubate them onto nothing so what’s the point of getting them on 5 on 5 because they’re obviously going to fail that, so…we might be like we need to put them on 5 and 10 because that’s more realistic as to what we’re going to extubate them onto, and then you would just write that in your notes. Some consultants are more strict and they’re no, it has to be 5 on 5, because it’s a trial and because of the research you have to do what that is, but some would be like well no, just write your comment…”* (PICU Bedside Nurse, S05)  *“If they think the patient might need non-invasive, because of what’s been happening for years, then they set up higher pressures. So we find some patients, they had high pressures, like five over eight or five over seven, and then they were still ventilated. So that doesn’t count as an SBT because…. after all the work, it ended up not.”* (PICU Research Nurse, S05)  *“And sometimes doctors are like "oh, they're not ready," and you're like "give us a better reason" and then you've got to put down "doctors have said not ready." That’s not a proper reason.”* (PICU Bedside Nurse, S15)  *“…I think there was always some issues about SBT in patients where the PEEP might have been slightly higher and clinicians didn’t want it dropped for the purpose of the SBT. Those little things in terms of protocol, but mostly I think it ran very well for the vast majority of patients.”* (PI, S06)  *“And, to be honest, most of the times we would have decided overnight whether we’ll be extubating or not, so we would just go ahead and do an SBT and that would be sort of extra information, or it would give us more confidence that child has passed an SBT and child is ready for extubation. As I said, the decision would be done the night before…So most of the decision will be done on the night before and it would be just sort of an extra precautionary exercise.”* (PICU Registrar, S06)  *“It’s a consultant thing as well. Our consultants aren’t a huge fan of it. But this has made it easier to put them onto pressure support, by having the trial, but I don’t think many of our consultants are a huge fan of putting on and leaving on pressure support because of the thing that they say “oh, they will tire.”* (PICU Bedside Nurse, S06)  **Sub-theme: Variability of consistency relating to unit routines**  ***Off-unit ward round***  *“Sometimes they would discuss it in medical handover, then it wouldn’t relate to the actual ward round…With the nurse in charge, if she or he is free, and then they do a ward round then sometimes it wasn’t spoken about in both. I think maybe because they thought oh, we discussed it, and it was lost in translation.”* (PICU Bedside Nurse, S13)  *“So what would happen is the file with all the documentation would go in. The nurse from the bed space goes in with the charts for the last 24 hours, the medical notes are there, the X-rays are available and the nurse in charge quite often, obviously, would be filling in the Sandwich details for each patient for every day. It was that process that wasn’t necessarily being done, and it wasn’t necessarily just the nurse in charge that wasn’t doing it, it was very rare that you would hear a doctor saying, “right, this is the comfort score we’re thinking of”…you know, it would be a the end of ward rounds somebody reminding the doctors to do those things.”* (PICU Physio, S18)  *“So obviously the nurse would go around in the morning, pre handover, down in our seminar room that the doctors do with the nurses and with other multidisciplinary team members, like physios, and then obviously that would be discussed down there and then when they'd come back and did each individual patient as a ward round, then obviously that would all be discussed…I think it was a case of you haven't got a choice… Because the [bedside] nurses were asking me and [name of another research nurse], or whichever nurses were doing the handovers would request that and the nurses wanted to know what their patient’s plan was for the day.”* (PICU Research Nurse, S17)  *“…that’s when decisions are made, in that handover around extubations, that kind of stuff. So that was where it needed to be discussed...But also, I think because we do the handovers away from the bedside. If that handover was done at the bedside then the nurse at the bedside might have more opportunity to be involved…The team leaders…So they were the ones to provide an update. I think it was challenging…to remind the team leaders to come with the correct information and to actually*  *volunteer that information without having to keep being reminded.*”  *“I think it helped with the discussions [but] I think there was always a barrier, so, it helped bring up those conversations about it, certainly the sedation, the weaning and stuff like that, but there was always this barrier between where our handover is held and those sorts of discussions and there was a lot more backwards and forwards…”* (PICU Senior Nurses, S01)  *“… before they even set eyes on the patient, they decide to do the ward round off the unit without the bedside nurse. So the nurse in charge is there, but there’s only so much you can tell a nurse in charge, because they’ve only got one head, one brain, they can’t retain it all. So I think in that case, so then they would come round and say, "oh, well, you know, they’ve been on SBT, you can extubate them," you'd be like "well actually, I’ve come on and assessed my patient and actually, no, I can’t."* (PICU Bedside Nurse, S07)  *“That was another thing that changed, because the ward rounds always used to be done at bedside, which was fabulous, because they were there at the same time as we’d all be doing our rounds and the physio was there and could put their comments in. Much more at that time the doctors used to ask our opinions, they’d ask, if we’d bagged the patient, have they got a leak, that sort of thing, and there would be instant feedback…”* (PICU Physiotherapist, S06)  *“And I’m the person that sits in the ward round and I’m not always sure that they really do discuss it. It's been more me piecing together the information.”* (PICU Senior Nurse, S06)  ***SBT screen/SBT***  *“I think as long as you get it in, in the morning before ward round, and probably the least busy time would be later on in the night shift. So at the end of your night shift, if you do an SBT, they pass and then you can hand that information over, they've got that ready for ward round, "Look they’ve passed the SBT." That’s the time when we extubate primarily, is on ward round, or just after. We won’t extubate later on in the day.”*  *The 4 p.m., we’d always know right, okay, so overnight we’ll wean for a planned extubation tomorrow … you know what you’re going to do with your sedation overnight and how you’re going to prepare your patient for the next day.”* (PICU Bedside Nurses, S06)  *“I’m just thinking it’s that engrained "we only extubate in the day" thing. It’s, to me, the biggest stumbling block. It doesn’t matter how good the patient is. There’s even a point in the afternoon where they’re like "okay, it’s after 5 o’clock, let’s not." Occasionally they will, depending on the patient, and that’s a real cultural change and I’m not sure how you change that culture.”* (PICU Senior Nurse, S06)  *“So yes, we did have set times, and on day shift it [SBT screen] would be 11 in the morning. It was originally five in the afternoon but then we changed it to three in the afternoon because a lot of patients were not being extubated because it was too late by 5 o’clock… and obviously if they passed it they were then put onto a spontaneous breathing trial … I think the morning one was quite effective, because you’re gearing up for the day, whereas the one in the evening…”*  *“… so generally what we'd do is then the 5 o’clock one [SBT], probably knowing they were going to pass it, but you never obviously know for sure, do the 5 o’clock one and then it just gives you the nightshift then, so by the time it’s dayshift, the nightshift have got them ready to extubate and the dayshift don’t have to do anything apart from wait for… usually around ward round. So at 10 o’clock is generally when they'd start to think about it, because ward round is done, they’ve been starved for four hours or so…”* (PICU Bedside Nurses, S13)  *“So it [SBT] was done at 6 am in the morning, and we’d do that one at 12 in the day… Six in the morning, I would say, was the better one… because you were having ward round in the morning at half past eight and if they’ve passed their SBT… Okay, extubate.”* (PICU Bedside Nurse, S10)  *“So they get [SBT] screen six hourly and depending on what type of patient it was would depend if they’d actually get an SBT. So we kind of pushed the six in the morning and midday, but physical SBTs, and I never discourage people, I'd say "if you want to do an SBT at midnight, you absolutely can, but you’re not going to extubate them at midnight."*  *“Six in the morning [SBT] … So you had all this information before ward round and you could say "right, we’ll finish ward round, let’s start extubating people," and you could start early enough then.”* (PICU Research Nurses, S10)  *“Not routinely no. We would never extubate overnight. Maybe in the afternoon [or] evening…”* (PICU Senior Nurse, S16)  *“If it’s [extubation] kind of been thought about for the whole day, but probably less so if it’s a bit of a surprise that the patient passes the SBT screen in the afternoon. I think a lot of people are then more likely to say well, we’ll just give them another 12 hours and do it in the morning.”* (PICU Research Nurse, S18)  *“I think the morning one worked best, because you knew it was coming up for ward round and you needed to do it. I think the one that they really struggled to get done was in the afternoon, more towards bedtime, I think they just got lost … so many things happen you either forget or there’s other stuff going on, so the SBT [screen] doesn’t take a priority all of the time.”*  *“I liked the morning one. I think the afternoon one, for me and my patients I had, it just didn’t tend to change that much. So we were always going to take the tube out or we weren’t anyway.”* (PICU Bedside Nurses, S03)  *“It [overnight extubation] depends on the patients. We tend not to extubate after midnight, if it is possible, but in some cases, there are post op cases where if there are [no] problems we do extubate.”* (PICU Consultant, S03)  *“Six in the morning, just because people wash their patient and sort everything out and then you think "oh, okay they’re going to extubate, let’s see if they’re ready," whereas at two o’clock in the afternoon they don’t really tend to be like oh, let’s take the tube out. They normally do it after ward round at 10 o’clock in the morning.”*  *“They'd do it at six o’clock [am] and then they would put them back on their normal settings and then extubate them at 10 or 11. So it was never straightaway, but they’d wait for their handover and do it later on.”* (PICU Bedside Nurses, S14)  *“…a lot of people would screen at 10 o’clock [pm] and do the SBT, but then be like “we’re not extubating tonight, we’ll do it in the morning”… But…I felt like people need to extubate them before the ward round for that 10 o'clock on. Maybe…seven o’clock [am]. I know it’s not great on a nurse but we’ve all done it and if it means getting that tube out of that patient then…”* (PICU Research Nurse, S14)  *“The screening was first thing in the morning before the nursing staff went off the night shift and then lunch time, and the reason we did that was that we wanted to try and aim for extubation basically within office hours. There are patients that we would extubate overnight, but generally you’d want to extubate during daytime, generally.”* (PI, S01)  *"See if they pass [the SBT] in the morning again." Very rarely I feel like they would extubate in the afternoon… they'll [doctors] just say "keep them intubated overnight, do it [SBT] in the morning and we'll see tomorrow." Yes, and understandably as well.”*  *“I've been here a year, I think I've done it [SBT screen later in day] about maybe once or twice, because like we said, they normally extubate in the morning.”* (PICU Bedside Nurses, S15)  *“… hopefully you'll get extubated before eleven, before ward round, but if they've done it at five[am], that's like six hours sometimes a gap, like in between if they have to do…there is a routine inside the unit and sometimes you don't break the routine, because either it's unsafe or people are not ready or things like that.”* (PICU Research Nurse, S15)  *“I think the other difficultly was six am you might put them on and then if they were fine, two hours is eight am and eight am was crossover time, handover time… and then the doctors are on the ward round or something.”* (PICU Senior Nurse, S15)  *“… but if it’s after that time [6pm] we’ll wait until tomorrow and let’s just gear him up tomorrow… we’ll wean … we’ll stop sedation at 4 [am]”, we’re nil-by-mouth anyway at 4/6, “and we’ll get going, first thing.”* (PICU Research Nurse, S07)  *“…because we go around at night and I actually did then find myself saying "and can I say that there’s no point in this child having an SBT tonight," but it had taken me a few experiences to realise that I had to say it.”* (PICU Consultant, S07)  *“Did we do six and six, was that the plan, but I do think the early morning one…”*  *“Was more the effective, I think.”*  *“…and it was more effective but I do think sometimes they left it till later because you’d got a change over of shift, and you do your SBT at six but actually we don’t have ward round until half nine/ten, and so we’re not going to extubate until generally after then anyway.”* (PICU Bedside Nurses, S17)  *“A little bit of confusion, because sometimes some of the more enthusiastic registrars would do the SBT at six in the morning and we'd sort of go back and say actually you shouldn’t be doing it, you should be doing it when we’re ready to extubate, which would be a bit later. But that was a couple of issues.”* (PI, S17)  ***Other activity***  *“Because it happens all the time, even if you take Sandwich out, we have children that they say “oh, we’re going to extubate on ward round in the morning, we’re going to extubate today”, and it doesn’t happen because practically there’s not the people around or they’re tied up with someone else doing something else that is more of a priority at that point.”* (PICU Physio, S03)  *“I think it depends on the time of the day and what else is going on in the unit. Otherwise, you normally would go ahead if he passed.”*  *“Yes, or there’s other multiple key unit activity that’s happening on other patients and therefore the staff wouldn’t be able to respond appropriately if there’s an extubation failure.”* (PICU Consultants, S03)  *“I mean, I’ve got to be honest, on more than one occasion I’d do the SBT and be like "yes, okay, my patient’s on 5 on 5 but actually… we’ve got to do this, we’ve got to do that, so we can’t extubate," and I found that happened on more than one occasion.”* (PICU Bedside Nurse, S03)  *“Big one, waiting for an MRI scan…And also sometimes the unit will just be too busy and too much happening at the same time and it’s just not safe to do it now, so…”* (PICU Consultant, S11)  *“… and then sometimes, you know, we might be ready to go and wean and extubate and they want to go and have their break and they’re covering. So breaks are a big issue and more junior staff maybe covering. So that slows us down a bit, I would say.”* (PICU Physio, S04)  *“It all depended on staff resources really and the time. We only have one registrar in PICU, so if they were busy with a procedure, if they were on a lunch break, if they were at the x-ray meeting or macro round then they would have to wait until someone came back.”* (PICU Research Nurse, S10)  *“I mean there’s extraneous factors, going to scan, going back to surgery and you think oh yes they could easily extubate but surgery is planned for tomorrow we’re not going to extubate and reintubate, that kind of scenario…”* (PICU Senior Nurse, S10)  *“Mostly to do with what else is happening on the unit in terms of logistics; like somebody else is being intubated, somebody else is being extubated, some procedure is happening, that kind of thing.”* (PI, S18)    *“It has to be done at a safe time. And then obviously with other pressures that are happening on the unit, if you’re getting multiple admissions, that takes priority. So you’re safer to stay on, if they're coping on CPAP, keep them on CPAP and you keep the tube in and then…”* (PICU Registrar, S02)  *“Especially at night-time, there’s only one registrar on. If there’s something full on in one bed, they just don’t have the time to do that. So even though you have to wean something, you can't do it really.”* (PICU Bedside Nurse, S02)  *“Not always, especially because the bedside nurses can’t change the ventilation because you’re waiting, and if something else happens or they then need to put in a vas cath into a really sick new admission who then might have to go on ecmo, you’re not going to see that consultant for at least three hours.”* (N/PICU Research Nurse, S08)  ***Fit with unit feeding & other routines***  *“For a few years now what we do is we stop all feeds at 6 o’clock for every child. So we only feed them over 20 hours anyway. So by the time you’ve done the ward round at nine, nine thirty, you’ve made the decision, you’ve got half an hour and they’ve had four hours of starvation.”* (PI, S17)  *“…once a child is on C-Pap pressure support… the position we take is “okay if you’re thinking about that [extubation], then you should be stopping feeds you should blah, blah, blah.” So I think what Sandwich actually [showed], because of the rapidity and quite successful SBT trials is that one of the reasons the patient doesn’t necessarily get extubated is because of that closed loop… you still haven’t stopped the feed, it's all those…So they asked about the SBT but there was no thinking about what happens after the SBT, it’s obvious we pull the tube out; if we’re going to pull the tube out see if the child is fed…which we are now going to stop the feed. We’re going to get the next…what’s the next interface, so that is the missing link that I have observed.”* (PI, S03)  *“We stop in the morning to plan for extubation. So we stop our feeds at six if we think we’re going to extubate that day.”* (PICU Senior Nurse, S16)  *“Well, we usually have to wait four hours from stopping feeding, so we usually then, if they managed on CPAP then we’d prepare them for extubation, but then you’d have to wait four hours, you have to stop feeding them.”* (PICU Bedside Nurse, S13)  ***Compliance aligned to perceived relevance***  *“One of the big hindrances with compliance to doing the paperwork, that I’ve seen, was either the really sick patients or the really long-term patients, and you’d go to the bed space and have a look, we’d go around each bed space every day, and often they didn’t see the relevance of doing the paperwork when they didn’t see their patient being at that point in time. I think they were much more compliant when "oh yes, actually, we’re ready to look at weaning, let’s do this…”*  *“The bedside nurses…they knew themselves that this patient wasn’t appropriate for the screening. But I think if they could have almost taken that job away from themselves, because I think they felt a bit frustrated at having to do it even though they knew it wasn’t going anywhere…”* (PICU Research Nurses, S03)  *“To start off with, there was some kids who were on long term weaning plans that weren’t being planned to extubate… we were doing it four times a day, but then…the research team came with stickers and every patient who was identified as a slow wean, we only had to do it once a shift, and I think doing it once a shift wasn’t an issue.”* (PICU Bedside Nurse, S03)  *“Yes, I think that’s also true because our unit has a lot of sick patients and long-term patients, because some of them are pretty obvious that it won’t be in our plan to do anything, you know. So it becomes irrelevant in some cases.”*  *“It just added to the work… the tick box added to this you know, and it makes people disengage.”*  *“I agree with that. It wasn’t really adding to some patients’ management at all, because there are patients where you may have decided we’re going to keep the tube…and therefore in those patients being reminded around three times a day as to do you want to extubate when we have said, no, no, for 21 days and therefore 63 times that you’re going to ask this question the answer is no. So that was an annoyance.”* (PICU Consultants, S03)  *“When they know that it doesn't look like they're going to be extubated, people kind of relax a bit with those patients… you'd need to go actively and tell them like "just remember to do your screening…"”* (PICU Research Nurse, S15)  *“… I know there were times… we were like "ah, it’s another form, we know this kid’s not going to do this. What's the point? We already know what the outcome is, I've got to fill out another two forms for this just to say no."*  *“I think it’s good for patients which are clearly like close to extubation, but you had to do it for everyone and if you'd got a patient that had got an open chest and they’re really unstable and like you’re really busy and then sometimes I would just do it as I’m about to go home, because it’s something obviously that’s not going to make a difference to that day, I’m busy doing other things, I’m only doing it once a night when the person’s arrived and then I’m filling it out just as a thing to fill out. So I think in that situation it’s a bit annoying, because you’re clearly not going to extubate with their chest open. I think it is good for reminding you to look though actually that they might be able to be extubated.”* (PICU Bedside Nurses, S05)  *“I feel like the attitudes of staff, so especially more senior staff who did tend to take the sicker patient just didn’t like Sandwich because it was extra work for them that was irrelevant to their patient, if that makes sense. So for them it was a bit of a pain in the bum. Having to make the time to do this paperwork that needed to be filled in and people were reminding them to fill in, that made no sense to their patient, but every day they had to do it, that didn’t make sense, and me going around the bed space and reminding them to fill it in, I felt like I was apologising, I was like "I’m sorry, I understand, but it’s…"* (PICU Research Nurse, S01)  “*I think we were probably all guilty…. but I think the doctors became fatigued by it because it was seen as yet another exercise on a ward round, that was frustrating. The team leaders were frustrated… they were having to try and put SBT or try and document to say why we’re not doing this on patients who it’s not eligible for, and that increased resentment even though it could have benefited …there would still be a discussion about their weanability because of the paperwork, even though it was ridiculous….it just felt a bit silly, so why would we even start the discussion of "are they eligible for an SBT?", their chest is open or they’re sort of aiming for these sort of comforts, Comfort B on patients that it's not applicable for, like a neuro patient or something like that.”* (PICU Senior Nurse, S01)  *“So that [SBT screen] was a bit that was less well performed. Sometimes the assessments would get forgotten, particularly if we knew that we weren’t thinking of extubating them, so they were being neuro protected or they were oscillated or whatever, they would miss out the assessment. And you’d say you’ve still got to do the assessment and say they’re not ready for an SBT. So that would tend to be less well done.”* (PICU Consultant, S16)  *“So there were times when screens were missed because people thought they didn’t need to do the screen because there was no plan for extubation. I don’t because obviously I’m not here 24 hours a day, but they may have asked one of the junior doctors do I need to do the screen, and they might have said oh, no, don’t bother because we’re not planning to extubate them… and I think that was an ongoing issue…so the patient wasn’t going to be extubated anyway because they were still paralysed or having surgery the next day or there was airway swelling or something, so they probably thought oh, we don’t need to bother…”* (PI, S16)  “*I found that out through trial and error rather than somebody saying to me that you don’t need to be doing that on that patient. So I think there was that frustration there that perhaps a large percentage of our patients, you don’t have to do it.”* (PICU Research Nurse, S14)  *“It was a bit frustrating sometimes, because it was obviously extra paperwork for us. So yes, of course I did, when you had other stuff to do…. We’re not going to do it because of this…" It’s just because you’re filling it in and giving the reason, you feel it should be the doctor giving the reason why the patient's are not extubated, not yourself.”* (PICU Bedside Nurse, S02)  **Sub-theme: Availability of appropriately skilled staff**  *“In terms of the middle of the night, we wouldn’t tend to, simply because of lack of skilled staff should extubation fail. That practice hasn’t changed, and I think it will take a lot for that to change, because that will mean senior cover overnight and how we'd provide it.”* (PI, S06)  *“I would say that the thing that didn’t happen, and I don’t see changing, is we may do a Spontaneous Breathing Trial at two in the morning if we sense that they’re ready, but that child will not get extubated until ten, until the ward round has been around and the doctors are here, and that’s a cultural change that I don’t feel has worked, and I don’t know whether it’s just resistance to that or a safety… I mean, there is a safety element to it, but even if a consultant was sleeping in-house, I still don’t think it would happen and the safety element would have gone then … we’d normally extubate in the morning after ward round. We’re not a unit that extubates in the middle of the night, generally, because there isn’t the specialised cover if we need to reintubate. So generally, most of our children are extubated… in the morning.”*  *“Yes, and I think, rightly so, it should be the final barrier, because you shouldn’t be taking the tube out unless you can put it back in. And I won’t let the doctors do that here unless I know they can do it. So this is the big barrier to everything. Anybody can take an ET tube out, anybody can do it, but not everybody can put them back in.”*  *“We have a number of nurses [ANPs] that have already fulfilled the criteria. … But they don’t currently work in that role, in that capacity.”* (PICU Senior Nurses, S06)  *“…although there are two advanced nurse practitioners here, based on what I have been told, or three. They don’t intubate, they don’t use their qualification in prescribing and that sort of thing.”* (PICU Bedside Nurse, S06)  *“I did have one where the patient didn’t need the tube for the night, but because of staff cover and no medics that could actually intubate at that point without a consultant coming in, the patient was left on a CPAP for five and five for the night and quite happily, you know, it just sat there…because, obviously, there might have been one done, but then it wouldn’t have been safe to actually take the tube out, because obviously you’ve got less staff, less people around should anything happen.”*  *“If they were probably quite agitated and it was safe to take the tube out, as opposed to letting them self-extubate, that may have been an option. Other than that, it would probably be a ward round when it’s safe and there’s more people around.”* (PICU Research Nurses, S17)    *“I think with the SBT a lot of it was... so overnight a consultant isn’t resident here. So then it would be down to regs to okay with the SBT, you know, changes of regs, as in, like a new set...”*  *“Yes, which obviously doesn’t help.”* (PICU Bedside Nurses, S17)  *“It’s one of the things where we fall maybe a little bit behind other units, purely because we don’t have 24/7 consultant cover…on site, and certainly that’s a twerk with us, and you will find our SBTs, the majority will be in the morning and then there will be the odd one in the afternoon or in the early evening, but certainly not, I wouldn’t expect that beyond 6 o’clock or whatever it is…”* (PICU Consultant, S17)  *“…I’ve got to the point where this patient’s ready to wean and just try and get someone as quickly as possible, otherwise you can wait hours before someone actually walks into your bed space...but I do feel like some patients passed the SBT screen, ticked all the right boxes, should have gone onto an SBT but there wasn’t the people around to put them onto the SBT at the right time. So they may have passed the SBT screen between 5 and 6 a.m., but they’ve not been put onto an SBT until 8 a.m. when the next available person’s been there to switch them.”* (PICU Bedside Nurse, S01)  *“I think there was a general reluctance to progress at night…Because the staffing I don’t think is there, the consultants aren’t necessarily there, the consultants, they don’t want to extubate at night. There’s numerous reasons for not extubating at night, aren’t there. There’s staffing, but it’s also that if you’re planning an extubation, that’s not the ideal time for a patient and their family…So in the morning just makes more sense, for lots of reasons.”* (PICU Research Nurse, S13)  *“I can’t speak for everyone, whether they were happy to do that, but I was shown how to do it [SBT], so I knew how to do it. I’d tell a senior nurse if I were doing it, but maybe for the ones who were new, probably the senior nurse would have done it for them.”* (PICU Bedside Nurse, S13)  *“Mainly because the people that have to change the ventilation onto an SBT is the Registrars and the ventilation management nurses, which tend to be the echmore nurses, filter nurses more senior nurses…firefighting… but obviously you need someone to enact this …”* (PI, S04)  *“I think sometimes some of the medics were unsure and would go, "well, we’d want to wait.”… so the junior doctors, the regs would want to have consultant overview on it.”* (PICU Senior Nurse, S04)  *“…at 6 o’clock in the morning, you’ve got two doctors who’ve been on for an 18 bedded unit overnight, so they’re generally trying to get ready for handover and things, whereas if you’ve got nurse ventilation management people on, we can go round and flip them into SBTS and get that bit done. And equally, I’ve had it quite a few times where the consultants let me know that there’s a plan to extubate these children, but… [a] very sick patient population has taken the doctors attention, whereas now we can move those children that are ready to move forward in a timely fashion.”* (PICU Bedside Nurse, S04)  *“… very rarely during working hours do the consultants have to come and extubate because there’s always me or some of the junior doctors around. So I wouldn’t have thought that [delayed extubation following a successful SBT] was so much of an issue…I couldn’t say for overnight because I am not here then.”* (PICU Physio, S04)  *“Our consultants are quite relaxed and they’re quite happy for nurse led extubation, but we usually need a medic to say okay, go ahead, but they’d be happy, as long as one of them was on the unit, for us just to crack on and do it… All the band sixes. Pretty much anyone that’s done a PICU course will have done several extubations, and we teach our Band 5s from kind of dot, but no Band 5 would extubate a patient without at least a Band 6 there* *… we had quite a few times, and again it involved re-educating the medics [registrars], they wouldn’t have a rationale as to why they wouldn’t let the bedside nurse put them on an SBT, and it was kind of re-education with that, saying "what’s your argument for not doing it?", because we have all this evidence to say they should, you’ve got nothing to back up why we shouldn’t, so we’re going to do it anyway, and that’s why we broadened the senior person that needed to be discussed with not just the medics, so it was the medics and the nurses in charge…* *because usually the consultants, some of them will be around a lot, some of them will just come forward round, be around for a few hours and then go…”* (PICU Research Nurse, S10)  *“So we wouldn’t necessarily electively extubate them at 3 o’clock in the morning, even if they’ve been on an SBT and they looked really well, because they’ve got a grade 4 airway and it wouldn’t be safe to necessarily do that at five/six in the morning. We’d want the day team in and we’d want it to be a bit more controlled. I think we have a lot of complex airways.”*  *“In my experience they were extubated soon after the SBT. Things that would delay it would be things like availability of the doctor or the availability of someone to potentially support a junior nurse that isn’t used to extubating, that kind of practicality.”* (PICU Senior Nurses, S03)  *“We’ve taken part in a Collaborative Weaning course…So now we’re able to actually change the settings on the ventilators, which… At the start of Sandwich, we hadn’t commenced doing that… And I think that’s probably the benefit of our unit, the nurses can ask and some of the physios can start to wean.”* (PICU Physio, S03)  *“… we do not have a rule that nobody gets extubated [after] 10 [pm]…but equally, if you have a unit that because the registrars are not airway trained or they need anaesthetic support, then at the end of the day, the safety of the patient needs to come in play...so if you decide the SBT is at 10 pm but then you don’t have anybody to extubate until seven in the morning, then is it really going to be good for the child to remain sedated all night long….which to be fair, we didn’t see as much in [name of unit] because we do…a 24/7 extubation, bar the children who have got difficult airways, we normally don’t extubate them in the middle of the night anyway, because if there was an emergency, you know, when you don’t have a consultant present … I was a little bit disappointed because sometimes with the lack of personnel etc. these children could be on five and five for a significant couple of hours before they actually get extubated. So I know it's marginal gain but one could also argue that I have also observed where some of the nurses are putting the sedation back up just to keep the safety rather than this child suddenly rip the tube.”* (PI, S03)  *“Like for instance, a classic example, we don’t extubate patients on a nightshift because of safety reasons, because of just kind of medical patients that we nurse and a lot of other decision making processes that we follow, and I still try to figure out how Sandwich would help us to do that on a nightshift. I couldn’t find a link…”* (PICU Ward Manager, S16)  *“I don’t know whether Sandwich could have done better, I think its more of how the units work in themselves, and in some units physios are more involved, and it doesn’t necessarily depend on the Sandwich trial but no one ever said to me “oh you can’t do that because of Sandwich.” It was just…no one’s ever done that before as a physio. So it’s more of a unit by unit, how they use physios, rather than Sandwich itself. You could always put in physios can also be involved… Yes, definitely. I think it’s a really useful thing to do, because at the Evelina there's more people* *that can extubate… I really noticed that difference, coming in, when there’s less of them.”* (PICU Physio, S16)  *“We are only… PICU trainees. So I think it's more to do with confidence, you know, because we would have always had to get a consultant in to supervise the way we extubated.”* (PICU Registrar, S02)  *“So, again, they wouldn’t extubate unless there was a consultant around or whatever, so that would never happen during the night. It would depend on the timings and what staff was available, who was around.”* (PICU Bedside Nurse, S02)  *“Not at 7 o’clock, and don’t start doing them at midnight, because nobody wants to extubate somebody at three in the morning. We adjusted the time when we did the SBTs to take that into account…once you thought about and put them in a different place, you know, that was…we just…[name of PI] and I thought about it in the beginning and changed the timings of some things.”* (PICU Consultant, S02)  *“The thing I think, it didn’t change our overnight practice that much, because my experience of what would happen is that the trainees would report in the morning that the patient had passed their SBT at midnight but their patient still wasn’t extubated. So if I was there and I drove the extubation and I said to them "right, let’s do the SBT now and I want you to extubate this patient, because I’m confident that they’ll be okay," then they would do it… Like I felt they would frequently hand over the patient, passed their SBT at midnight…passed (SBT] at 6[am], but they wouldn’t have extubated them.”* (PICU Consultant, S11)  “*Or the ANPs would extubate them in the middle of the night but then the trainees wouldn’t.”* (PICU Consultant, S11)  *“…then we could then change the ventilator setting to C-Pap pressure support ventilation, so five on five, and then sort of wait and see. So it purely depended on, I think in the end, how confident or how experienced the bedside nurse was…If the nurse felt confident to and was an experienced senior nurse, they could then… obviously you'd run it past the doctor before you did it...”* (PICU Research Nurse, S14)  *“I think because [not extubating during the night] sometimes there’s not a consultant around, or sometimes it's like really junior staff that aren’t confident in extubating straightaway ... I don’t know if it’s just common practice to do it in the day.”* (PICU Bedside Nurse, S14)  *“Like for instance, a classic example, we don’t extubate patients on a nightshift because of safety reasons, because of just kind of medical patients that we nurse and a lot of other decision making processes that we follow, and I still try to figure out how Sandwich would help us to do that on a nightshift. I couldn’t find a link…”* (PICU Ward Manager, S16)  *“…there was no point in it most of the time, because you’ve gone through the screening but actually there’s nobody there who could do it, or they'd come and do the SBT and then you wouldn’t see them because they’re down the other end of the unit…it’s not through anybody’s fault.”* (PICU Bedside Nurse, S07)  *“I think the reason we probably were interested in the Sandwich stuff was because we do, do quite a bit of weaning on the ventilators, me and my other colleague who’s in a band below me. It’s taken quite a while to get our consultant colleagues to agree to that. But we do, do quite a bit of weaning. Perhaps we’ll wean more readily than the trainee medics do, because we’re here all the time, a bit like the ANPs in that respect.”* (PICU Physio, S18)  *“I just think the timing works. In the morning that’s when you look at your daily plans… The bedside review in the morning, because that’s when you kind of review everything. I think it’s a good time to…like we do the screening in the morning and then know going into ward round whether it’s something you can say they’ve passed their SBT screen, can we try them on CPAP. So you’ve got everyone there then …”* (PICU Bedside Nurse, S18)  *“And I think the other thing you’ve got to take into account currently within this unit is that we have been two consultants down. So they have limited off time, they have work that isn’t based on this unit as clinical clinicians, and you’re asking an awful big change at a time where they are high-pressured work and time poor…. “So I think like the whole of the NHS currently, higher staffing numbers would help us with this. If were at full complement of consultants, there’d be a better chance that they’d be willing to be in-house, but we’re already robbing them of a lot of time with their families.”* (PICU Senior Nurse, S06)  *“And then also it can be sometimes if people have just simply forgotten or if there’s been a change in staff from the medical team or consultant team, it’s [SANDWICH] not been mentioned as much, they haven't bothered about it.”* (PICU Research Nurse, S08)  *“So we’ve had a lot of locum consultants recently. I don’t think that probably helped, because they always have different ideas, regardless. I think our main consultants mostly sing from a similar hymn sheet, mostly.”* (PICU Bedside Nurse, S17)  *“Yes, we’ve got quite a junior skill mix in our unit at the moment and we’ve had quite a lot of new starters over the last 12 months, and I think sometimes with the more junior nurses, it was the same as anything that’s kind of new to practice, it was, for them, having it on their time management radar, if that makes sense.”* (PICU Senior Nurse, S04)  *“I felt it was the new nurses and the really been here a long time nurses that didn’t pick up. The ones who were kind of more adaptable, in the middle, just took it on and did it.”* (PICU Clinical Educator, S04) |
| **Theme 5: Promotes inter-professional working**  *“On the sedation part, I thought that was quite helpful in focusing the whole team’s approach to sedation. I thought that the groupings, if you like, and the colour coding and being able to discuss if they were in the green, for example, was quite helpful… I think it did improve the focus everybody, but particularly the bedside nurse on sedation, and give them some additional empowerment to change things.”*  *“I would agree… that actually getting that focus on sedation scoring and getting a consistent approach to what we wanted was really helpful… to make sure the children are comfortable and safe when they’re ventilated, but also not to impede extubation when they’re ready, and getting that balance right, I think has always been a challenge and I’m not sure we’ve always communicated it very effectively before. So I think the whole point of actually having a coordinated approach where we can discuss it was useful, and using the comfort score consistently was helpful, and actually, not just using it but actually understanding it, more important.”* (PICU Consultants, S07)  *“… I think doing the screening was a perfectly reasonable intervention, because the bedside nurses didn’t necessarily have the understanding to know whether the children were ready to extubate, but they could easily do the screen and then ask someone else to do that.”* (PICU Consultant, S07)  *“In the sense that everybody was reading from the same page. Nurses are certainly more empowered to wean sedation and act on it, and that way things, I noticed, were moving forward quicker… when I came for the ward round, the team all night, which includes the nurses and the doctors, had already done an SBT and then they were already thinking forwards, which was good.”* (PICU Consultant, S07)  *“…because sometimes you can be there with a patient and you’ll be saying all day, "this child is under-sedated, this child is under-sedated, this child…" and they don’t listen to you, and then you say, "well, my Comfort B score is…And they’re like, "oh, okay, we’ll do something about that."* (PICU Bedside Nurse, S07)  *“So you felt like you were prompting them a bit more instead of the registrar coming around in the morning perhaps and saying, “oh, actually, they might extubate today”, it was you saying, “oh actually, their SBT… or, actually don't put them on an SBT because they’re still over sedated…””* (PICU Bedside Nurse, S07)  *“… and this almost gives us like a bit of an insight into how we make these decisions and it almost makes it feel like a bit more like teamwork.”*  *“Yes, because it's a lot less of a personal view as a goal you can actually reach and everyone can agree that, yes, they passed that.”*  *“I do like [that] they listen when we say they passed it or they failed it, they do listen to that decision. They don’t usually go, "oh, well, let's take the tube out anyway."*  (PICU Bedside Nurses, S15)  *“But interestingly, we have the "actually he looked fine at the SBT," so people started using the SBT as a tool to say "the SBT went off well so I think extubate." So that it started getting into a jargon as well, slowly.”*  *“I think the one thing we should take away is the nurse involvement in weaning… because that makes everybody avail of it, it's not just the consultant who's coming in every day and saying "I think we need to extubate … And this gives a certain amount of objectivity that we need to move forward.”* (PICU Consultant, S15)  *“I think it helped me crystallise my thoughts about why I didn’t want to do something, because sometimes you just had this gut instinct, this doesn’t seem like a good idea, and maybe before Sandwich I would just say “no, let’s just sit tight overnight.” But it made me have to crystallise. On the evening ward round, for example, “I don’t want to proceed because...”, and I think that was good in terms of shared understanding at the bedside.”*  *“And I liked that it empowered our nurses much more. [Our nurses] by definition are quite a forward pushing… as it should be, but I really liked that it gave them that structure of this is what we’ve done, this is where we’re at and challenged us to think, yes, there isn’t any reason why we can’t do this … It helps you to articulate it as well. This is what the guideline says but I did it differently because of this clinical judgment, and then you can explain it.”* (PICU Consultants, S03)  *“I think it was shifting a bit faster. I think it empowered…we'd always that cohort of senior experienced nurses who were collaborative weaners and could crack on with it, we’ve had more junior nurses coming through, so I think it helped to empower them to take it forward, and I do think it hopefully made and will continue to make us special in terms of mixed sedation.”*  *“And empowering nurses was also good in that they could then use evidence to say “well, this patient can be weaned, or not”, though to an extent that depends on the seniority and the experience of the nurse.”* (PICU Consultants, S03)  *“I liked that it introduced a bit more of a conversation between the bedside nurse and the consultant about the ventilation plan.”*  *“I'm a new nurse so this was just being put into place when I started…I think I’d definitely have the voice to say "this is Sandwich paperwork," but if they were like "oh, no we don’t want put them to 5 and 5, we want to do it the old way," I don’t think I would have the confidence to challenge that.”* (PICU Bedside Nurses, S03)  *“I think the nurses, the medics, and from our perspective as well we’d question them, why are they not weaning, and if they [patients] were static where they were, I’d go “have they met the criteria for this morning. Should we consider putting them on Spontaneous Breathing Trial … And we definitely adopted the language of SBT. That wasn’t used prior to Sandwich. It was "oh, we'll stick them on the bag, give them a feel, see how we go", whereas now it's “they’ve had [the] SBT” or “we’re planning an SBT for that patient … So I think that as a reference tool [the protocol] is really good because it stopped it being a conversation as to “my gut is I think they’re going to do or they’re not going to do”, this is the objective markers which suggest that they should do and therefore we should try, rather than trying to negotiate and say “well I think they will do.”* (PICU Physio, S03)  *“… it wasn’t carried out all the time but I think some of it, although it's dependent on clinician, I think it was dependent on the nurse as well and how confident she felt asking about the Sandwich or going ahead with the Sandwich, because sometimes during ward round the consultants would forget to address the Sandwich stuff, but not all the nurses would voice that actually we forgot this … the junior nurse probably would have thought it's not up to me…and they’d wait to be told, on the ward round that’s what they were doing, whereas now they can start to think…have that thought process before the ward round comes and then provide that information.”* (PICU Senior Nurses, S03)  *“… staff that are more experienced … and are a bit more vocal probably didn’t have that problem anyway escalating to their medic if they feel like they need to make a change, but I feel like for more junior staff it did help them to feel like … this patient does need this and I can use this to back up why I’m asking for a certain change to be done. So I feel it did really support in terms of giving the bedside nurse a bit more of a voice if they didn’t necessarily have one before or feel confident enough to explain why they want certain things done.”* (PICU Research Nurse, S03)  *“I think it’s taken out some of the consultant’s individual variability on a shift, so that’s helped.”* (PICU Consultant, S18)  *“I think it helps in handover in the morning, because the nurse in charge will ask the questions of the bedside nurse and it makes everyone in the room think it about it now, I think. If they have failed the SBT it’s then a discussion about what can we do, why have they failed. I think it does open up a bit more discussion on what’s the next step, do they need a little bit longer, could we wean their pressures down more steadily.”*  *“… especially in handover when the doctors are talking about weaning the pressures, whereas sometimes they wouldn’t even tell you, they would just come and wean without you knowing sometimes, I think. They’ve changed yes. So I think sometimes having that conversation if they have failed the SBT…”*  *“…it gave me the tool to at least be able to trigger the question and say “I think we’re under sedating or over sedating at this minute in time.” So again, it just gave me a really useful evidence base to at the very least ask the question whether we need to increase or decrease…. It’s the sort of thing that you’d run by a doctor. You’d say "actually this patient is this well sedated, or not, can we do something?" They’d say yes or no. So it's not ultimately our decision but we’d be the people suggesting it, yes.”* (PICU Bedside Nurses, S10)  *“… because I thought the Sandwich trial was absolutely brilliant, it really helped inform us of what was going on, we felt we were more part of the team as well…I think it was just the fact that we could immediately see, because we learnt more about the sedation scoring and what have you… but suddenly we had that information, we’d learnt about it a little bit…”* (PICU Physiotherapist, S06)  *“Everybody’s more aware… as a nurse in charge you can ask someone what is their comfort score and they know and they know what it means, whereas before they wouldn’t know or they would tell you a number, they didn’t necessarily know what that number meant. So it’s a better tool now.”* (PICU Senior Nurse, S06)  *“The thing is, it meant that the nurses would tell us this patient has passed his SBT overnight and therefore would you consider him for extubation. So it encouraged the nurses to raise the issue of extubation with the medical team. We all have different parts in the puzzle, so I don’t know that it matters too much. They will do the bedside test, they will tell us, we will make a judgement and discuss it with them and then we will all work to extubate the child in a timely manner, if it's appropriate.”* (PI, S06)  *“I think that Sandwich offered a systematic standardised approach in how we approach extubating a patient … it hastens the process instead of having to wait on the medical staff and therefore your nursing staff could be utilised in a more productive way.”* (PICU Bedside Nurse, S06)  *“…it definitely got us talking more and thinking, because that’s four times…well, at least on a 12 hour shift, twice, when you’re thinking on that shift whether we could extubate or not. So yes, I think communication definitely improved.”* (PICU Bedside Nurse, S13)  *“I think it definitely is working, because it is making everybody think along the same lines. Ventilating for too long is not a good thing and as soon as somebody’s ready you do need to take the tube out, and when these things were not there it was a very consultant dependent led thing. So there would consultants that were like "okay let’s give it more time … and then let’s do it." Then someone will come in and "oh, let’s take the tube out, we’ll see, we’ll manage, we’ll manage with non-invasive," but this gives more of a common ground where everybody is talking in the same tone. So I think it’s definitely contributed positively … having common words that we can use, SBT readiness, is really helpful and it makes communication more effective, efficient. So, I found it positive.”* (PICU Registrar, S13)  *“Why do I think it’s important, because I think there was still a tendency for us not to be speaking the same language. I think our aims might have been the same but I don’t think we necessarily had an easy way of communicating what we wanted to achieve. So if it provides a simple language, you know, this patient’s amber, this patient’s green, this patient’s blue, then that’s easy for everybody, and if people understand how that fits to a comfort score, great.”*  *“…I've had bedside nurses come up to me and say "are we going to do an SBT in the morning?" Yes, we are or no, we’re not, and the reasons why. Or "we've passed the SBT, shall I prepare to extubate?", which is really good, because it empowers and enforces, which otherwise they might not have considered or felt that they haven't had that empowerment to challenge, because they haven't had the tool, not to say that the culture isn’t there, but they didn’t have that framework. And so it allows them and encourages them to challenge what the decision making process is. So overall, my experiences have been really positive, from that point of view.”*  *“I think it's always dependent on the grade of nurses and their confidence and ability, and that's going to be variable, depending on personalities….this gave them [nurses] a structure, it allowed them a basis as to why are we doing, and if this was removed how many bedside nurses would say how much more are you going to wean the ventilation or move towards extubation.”* (PICU Consultants, S04)  *“… involve them in the decision process and to make them proactive in the raising that children were ready, and this has not happened… Not really, because I just keep on asking them, "have you done the…?" So you had to prompt them, so if I was to go and say "have you done the bedside checklist?", and they’re like "oh no, I’ll do it now. Passed! Great, what shall we do now?" So it had to be very led by us. So my registrars have also been, you know, going round and doing the SBTs, but they have been doing it, prompting the nurses to do the checklist, approving and then going forward, which kind of deters the whole point.”* (PI, S04)  *“I feel like we can say and we’ll be taken seriously and it will be questioned, but I do think that when it comes to communication at the bedside, it’s not the best because of things like, I don’t feel like we’re communicated with as much, I feel like the medics don’t tell us, where we will tell the medics pretty much anything… and I feel like it is listened to but then there’s a lot of things that change randomly and I’m not told about it. So a lot of that sort of stuff, it does get frustrating, doesn’t it? A plan is changed or if a drug is changed or if they've weaned ventilation, like you said, and not maybe told you.”*  *“You find the doctors will come in and change ventilation without telling me and I’ll just look at my ventilator and go "ah, when did that happen?" And if I have an SBT, I don’t know when it’s started.”* (PICU Bedside Nurses, S04)  *“Not that I have any bedside participating role, but from what I could see when I was in the bed space, everybody spoke the same language. You know, there was some sort of common themes that came out about the assessment and the SBT. Shall we proceed, you know? Is this a candidate, you know.”* (PICU Ward Manager, S16)  *“Consultant-wise, we’ve always wanted the nurse to be more involved in ventilator weaning than perhaps they have, and this has certainly helped it. There’s previously been a little bit of resistance in that some nurses feel that they ought to have done a ventilator module and they ought to be interpreting all the blood gases on their own and all that kind of thing, which probably they ought to be, but we have never managed to get that up and running. So we’re not a unit where nurses autonomously do blood gases and wean patients on invasive ventilation, so maybe this is a way to get that conversation going, and I don’t know.”* (PI, S16)  *“But also, if a junior nurse will say something at one time and get shot down, they will never speak about it again, and I think consultants need to recognise that if a tool that the unit is supposed to have adopted is being spoken about, then they should honour it.”* (N/PICU Research Nurse, S08)  *“The pluses on it was that its intention was to give the nurses more autonomy in the decision-making process and to give them an impetus and push get them more involved and not let things slide. I think that that worked up to a point. I think we didn’t get maybe as much of a sort of autonomous thing with all the nurses as we maybe hoped for in the beginning, but I think it did change their behaviour and did change their interest in talking to us about ventilation and stuff. I think in particular the younger ones, it gave them something that maybe the older nurses or the more experienced nurses already did.”* (PICU Consultant, S02)  *“I probably did find that it made the nurses maybe tell you a bit more about getting involved about how sedated their patient was, and if we were on the ward round and they said, "right, we’re going to switch off that," give them maybe a wee bit more confidence to say "actually, I think they need to be this". I think it involved them a bit more in the conversation in the ward round … I think it was good and it raised awareness of sedation… I don’t think we always did the right thing. But I think it certainly raised awareness in a multidisciplinary way, which was good… it ironed them [inconsistencies] out, in that everybody asked the question. So you were seeking the question everyday and you were looking at sedation, so that was more the sort of differences was ironed out, but whether you then went on to produce exactly the same result, no, I don’t think so.”* (PICU Registrar, S02)  *“But I think Sandwich is actually good in that way for extubation, because before that it was all consultant based, if this is the consultant that was on you would know whether the child is going to or not going to, but Sandwich has made that more consistent.”* (PICU Bedside Nurse, S02)  *“… because it gave everybody a structure on what to do when you come to extubation, isn’t it? Instead of waiting for the consultant to sort of say the magic word, it was all sort of lined up to be done … I liked the idea that it was presented as a quality improvement project, as opposed to a research project per se. So everybody was signed up to that. The more things that we have that allows the bedside nurse take greater autonomy over the patient has to be better… so North America bedside nurses do a lot more in terms of decision making as well. I don’t see why our nurses can’t do it here.”* (PI, S17)  *“… I think there’s a slightly challenging culture here for hierarchy, for speaking to hierarchy. Now, the consultants are very approachable. We’re quite fortunate, the consultants are very approachable, but there is sometimes a bit of a chain of command…some of the other team leaders are not necessarily approachable…if they didn’t buy into that [SANDWICH], then they would say [to a bedside nurse] "oh, I wouldn’t bother talking to your doctor about that. I wouldn’t take that any further," and that’s then a break in the chain of communication and so then they would be less likely to…. I think that’s more of a culture here that’s the problem…”* (PICU Senior Nurse, S01)  **Sub-theme: Final authority rests with consultant**  *“So I think, from the whole point of view, I think it's been really good for the nurses, it makes us want to continue, it makes us want to go on, and we still do SBTs, now the Sandwich trial is finished. But now it’s hard, because I'm not sure if I’m covered to do SBTs now because I’m not supposed to touch a ventilator in this Trust. Do you see what I mean? So now we’re at that point where we want to move forward and bring this into part of our practice but we need doctor support to do that.”* (PICU Senior Nurse, S06)  *“The consultants were really on board with the study and really drove us to try and do it, and also the doctors would come round and they’d say, "well, we have to at least see if they’d like to try." So yes, I think the consultants were really on board, and the research team would come round and double check.”* (PICU Bedside Nurse, S13)  *“… the consultants that were engaged, of which I would say it was definitely two, possibly a third, it worked really well, but if you don’t have ownership at every level, I think it’s more set up to [fail]…”* (PICU Senior Nurse, S10)  *“For me, I think it depends on which consultant. For example, I had two separate days where I had two different patients, they were both being extubated, and there was two different consultants on both days and one was saying "they passed the SBT there’s no point messing around, take the tube out," and the other consultant, the patient had passed the SBT but in their mind the patient wasn’t awake enough and wanted to wait for the patient to wake up more first. So that was very much sticking into their old way thinking.”*  *“… it did depend who was the consultant on. Sometimes they would do similar things. So if they weren’t awake enough, they’d pass the SBT but they were looking for other things to say okay they’re not, and then sometimes they didn’t follow it and just said okay, the tube can come out now. So sometimes it wasn’t followed exactly…”* (PICU Bedside Nurses, S10)  *“Certainly, with some of the consultants, if they then glossed over it, that empowerment is completely lost, but then even as more experienced nurses, we were getting disempowered anyway because they just didn’t seem to be that interested in that particular aspect at that moment.”*  *“I think the ward round part of it, I had very differing experiences, depending the consultant that was on leading the ward round…I think the consultants had varying levels of interest. I mean, if there’s people interested in research then they were fully throwing themselves into it, other ones, you’d really have to encourage them to even use it.”* (PICU Senior Nurses, S03)  *“… and what has helped it to work is having people who have influence in the area, who champion it. So, our medics, we found that bedside were much more compliant when the medical team going on ward round were prompting for it to be done or asking if it had been done. So we found a lot of the time, when that had been done, stuff was completed…”* (PICU Research Nurse, S03)  *“… there was a couple [of consultants] who were very, very good and very active and "ooh yes, thank you for the reminder, by the way we need to do this as well," and then there were others who were like "yes, it’s fine."*  *“I’ve got to be honest, I found you knew with certain people you had to push them harder to get them to do it…”*  *“… it’s not that they didn’t understand what they were doing… it’s that "it’s not my baby, I’m not really that interested, I need to get on to do other things." So that’s the kind of feeling that I got from it.”* (PICU Bedside Nurses, S03)  *“I mean, we were fortunate, we didn’t have anybody who was opposing. They [senior medics] had levels of interest and disinterest and neutrality… But there was no one who was in opposition, and we were fortunate, it would have been difficult if we’d had people in opposition.”* (PI, S18)  *“But one of the consultants started doing, was it seven or five or something else, I don't know what it was, but to wean them a bit more gently… that’s what he would have weaned, then he would have put them onto a higher CPAP setting and then weaned them down again if they felt they were open. “And other times, the other day a bronch patient just got two hours of an SBT and then went back onto the ventilator settings, didn’t continue, even though she passed…It depends who's on, because your consultants are not here all the time. Everybody likes different things, does different things.”* (PICU Bedside Nurse, S02)  *“One of the things we try and do more of here, and we can, is to allow the bedside nurse greater autonomy in making decisions, making changes, and some of them, they know we support them and they’re happy to do it. So this felt like a logical extension of what we want to do in this unit.”* (PI, S17)  *“He’s not here anymore, there was a locum consultant that we had that would follow the flow of Sandwich but would pick whatever numbers he jolly well pleased…they knew that [SBT settings]… because I put that many posters up it was in everyone’s face…I’d probably got three of our consultants that would follow it to the tee… and then a locum would just decide no, I'll put it on a Peep of six, but I’m not going to tell you why.”* (PICU Research Nurse, S17)  *“Well, one of the consultants was pretty anti it, to speak frankly, and didn’t believe in it, and I think that then sowed seeds for other medics who think "well, if they think it's ridiculous well why would I bother?" And then that permeates down to other people as well.”* (PICU Senior Nurse, S01)  *“… as far as I’m concerned, when I take care of my patients on the unit, it [SANDWICH] hasn’t made any difference, because my criteria for weaning and extubation are my own and I go around patients and turn the knobs and I do it all myself; and Sandwich or no Sandwich, I just turn the knobs and I extubate on the basis of criteria that are not just about how the ventilation is weaned but also how the patient behaves and is awake and all these things…”* (PICU Consultant, S16]  *“…but they would just override it anyway and would say, “I don’t want to do it”, so, for example, the SBTs, we did have a handful of consultants that … say, you're on the night shift and you do it [SBT screen] at 5 or 6, they’re ready for an SBT, "Oh, don’t do it, we know they’re fine." So it was kind of like, well...”*  *“Or it would be like, "Oh they’re too little, they won’t tolerate an SBT, they won’t tolerate going on CPAP. So actually, no, we’re not going to do it." It’s like, "well, why have I spent my time doing this SBT?"* (PICU Bedside Nurses, S07)  *“I think it in a way it did give more autonomy and more independence to the nurse to say… I’ve done my Comfort B, you know, they’re adequately sedated or… my SBT is failing because they’re over-sedated or something like that, but I still don’t think that maybe the medics would trust...”* (PICU Bedside Nurse (PICU Senior Nurse, S07)  *“Yes, our nurse educators were absolutely key with that, without them we would never have got through it all. We really just all pitched in. So I trained people, [name of another consultant] trained people, people were trainees, we trained people and we just shared it out amongst us.”* (PICU Consultant, S11)  *“I think people sort of didn’t change, and I really wished the doctors had pushed it a bit more. I felt like it had to come from them a bit more, because I kept saying like it’s a really safe way, like you know if you’re really not sure then put them on this, and you know, if it doesn’t work then you’ve still got the tube in.”* (PICU Research Nurse, S14)  *“Consultants. And if they think it’s a waste of time, for the whole week that they’re on you won’t hear it being spoken about once, and even if it is spoken about, it will be very passing. You really see a difference if a consultant’s not behind it.”* (N/PICU Research Nurse, S08)  *“… there was a consultant that was giving me a lot of trouble and then I had a personal chat with him and saying "you're making my life harder…because of what you're doing," and we are quite friendly [with] each other, [after their chat] he always does an SBT on patients and he always comes and tell us.”* (PICU Research Nurse, S15) |
